# Nanocomposite Strategy toward Enhanced Thermoelectric Performance in Bismuth Telluride

**DOI:** 10.1002/smsc.202400284

**Published:** 2024-08-08

**Authors:** Hua‐Lu Zhuang, Jincheng Yu, Jing‐Feng Li

**Affiliations:** ^1^ State Key Laboratory of New Ceramics and Fine Processing School of Materials Science and Engineering Tsinghua University Beijing 100084 China

**Keywords:** bismuth tellurides, energy filtering, interfaces, multiple scattering, nanoinclusions, nanocomposites

## Abstract

Bismuth telluride‐based thermoelectric (TE) materials have been commercially applied in near‐room temperature refrigeration. However, enhancing their TE performance remains crucial for expanding their application fields. Nanocomposite strategy has been widely reported as an effective approach to improving the TE performance of bismuth telluride‐based materials. In this review, the nanoinclusions are categorized into different groups, including nonmetallic hard nanoparticles, metallic nanoparticles, compounds with low thermal conductivity, and low‐dimensional materials. A comprehensive overview of relevant researches and present typical cases and recent advancements is provided. It is worth noting that nonmetallic hard nanoparticles are most widely used for reinforcing bismuth telluride‐based materials; the noticeable enhancement can be attributed to the interfaces that induce phonon scattering to reduce lattice thermal conductivity as well as multiple scattering effects along with energy filtering to increase the Seebeck coefficient. Although there exist challenges in terms of interface characterization and dispersion improvement for nanoinclusions, it is undeniable that the nanocomposite strategy offers a viable pathway to enhance the TE performance of bismuth telluride‐based materials. Therefore, further exploration in this direction is warranted to promote the development and application of TE technology at near‐room temperature.

## Graphical abstract

Nanocomposite strategy proves to be highly effective in enhancing the thermoelectric performance of bismuth telluride‐based materials, owing to the synergistic effect on charge carrier and phonon transport. This review comprehensively discusses the impact of various nanoinclusions on thermoelectric properties of bismuth telluride‐based materials, highlights the existing challenges, and presents promising prospects for future development of this strategy.

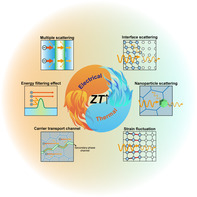

## 1 Introduction

Thermoelectric (TE) technology facilitates the direct conversion from thermal energy into electrical energy, representing a classical technique for energy conversion that has long been the subject of significant attention.^[^ ^1^, ^2^ ^]^ The TE performance is typically evaluated by the figure of merit (*ZT*). The rapid advancement in productivity and subsequent surge in energy demand, coupled with concerns regarding fossil fuel scarcity, has generated high expectations for TE technology in power generation. However, the progress in applying TE technology to cooling has been sluggish over the decades.^[^ ^3^ ^]^ In recent years, with notable advancements in optoelectronics, thermal management during information transmission has gradually gained prominence.^[^ ^4^, ^5^ ^]^ TE coolers, leveraging TE materials renowned for their absence of mechanical vibration and ease of miniaturization, have emerged as one of the efficient solutions.^[^ ^6^, ^7^ ^]^ The increasing demand for cooling applications further underscores the significance of TE materials.

Given that optoelectronic devices typically operate at temperatures close to room temperature, it is crucial to utilize materials that exhibit excellent performance within this temperature range for the fabrication of Peltier devices. Despite extensive research efforts being made to seek new TE materials during the last few decades, the alternate surpassing bismuth telluride in terms of near‐room temperature performance has not been found yet. In order to satisfy the ever‐increasing demand for room temperature applications, tailoring the TE properties of promising candidate materials within this temperature range has become a research hotspot in recent years. Although some materials such as Mg_3_(Sb,Bi)_2_,^[^ ^8^, ^9^, ^10^ ^]^ SnS,^[^ ^11^ ^]^ and SnSe^[^ ^12^, ^13^, ^14^ ^]^ have demonstrated superior performance at near‐room temperature, bismuth telluride‐based TE materials still rank at the leading level with unparalleled advantages in terms of reliability and industrial production.^[^ ^15^, ^16^, ^17^ ^]^ Therefore, further research on enhancing the TE performance of bismuth telluride‐based materials represents the most direct approach to meeting cooling application requirements.

**Figure 1:**
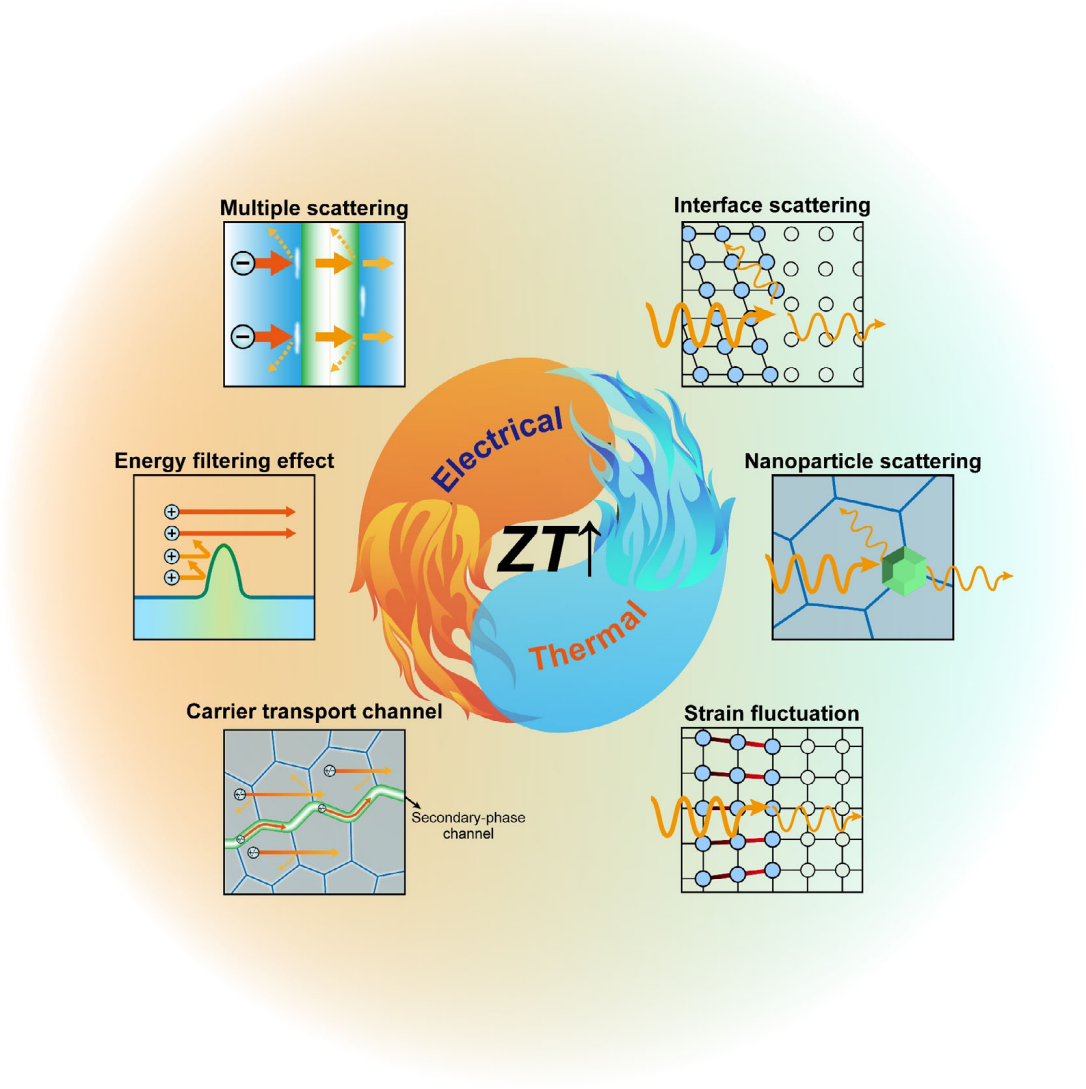
Schematic diagram of the enhancement mechanism by the nanoinclusions in bismuth telluride.

Although bismuth telluride‐based materials are widely recognized for their exceptional TE performance, the improvement of their mechanical properties has presented a persistent challenge because the weak interlayer gaps of the layered structure make them susceptible to cleavage due to van der Waals forces.^[^ ^18^ ^]^ To meet application requirements, the focus of research has been on polycrystalline bismuth telluride in the past 30 years.^[^ ^16^ ^]^ It has been discovered that fine‐grained bismuth telluride can surpass the TE performance of larger‐grained or single‐crystalline counterparts, particularly for p‐type bismuth telluride.^[^ ^17^ ^]^ This improvement is attributed to numerous nanostructures generated during the fabrication process, including nanotwists, nanoinclusions, and nano‐defect clusters, which greatly enhance phonon scattering and reduce lattice thermal conductivity (*κ*_L_).^[^ ^19^, ^20^, ^21^ ^]^ Among these defects, nanoinclusions have garnered significant attention due to their profound influence on both the thermal and electrical transport properties through different mechanisms such as the energy‐filtering effect and interfacecarrier scattering effect,^[^ ^22^, ^23^, ^24^ ^]^ as schematically illustrated in **Figure** **1**. Furthermore, during the fabrication process of polycrystalline bismuth telluride, the deliberate introduction of nanoinclusions can be achieved through various approaches such as direct addition or in situ formation. Different methods have been employed to introduce diverse nanoinclusions with distinct morphologies and physical properties, resulting in varied impacts on TE performance.

This work aims to critically review the current research progress in utilizing nanocomposite strategy for enhancing the TE performance of bismuth telluride‐based materials. The categorization of various nanoinclusions for bismuth telluride‐based composites is clearly presented. We provide a comprehensive overview of the present research trend, highlight the representative cases, analyze the key factors influencing TE performance, and summarize the existing challenges as well as future research directions. These findings can serve as a valuable reference for researchers who are devoted to investigating nanocomposite strategies to enhance the TE performance of bismuth telluride‐based materials in the future.

## 2 Strategies to Obtain Bismuth Telluride Nanocomposite

This review focuses on the synthesis of nanocomposite effects in bismuth telluride‐based polycrystalline bulks, where nanoinclusions are introduced at the grain boundaries of the bulk material. Depending on the different approaches employed to generate these nanoinclusions, the methods can be broadly classified into ex situ and in situ categories.

### 2.1 Ex Situ Nanocomposite Strategies

Ex situ nanocomposite strategies involve synthesizing the nano‐additives in an external environment. The key to incorporating nanoinclusions using this method lies in uniformly mixing the additive and precursor powders, guaranteeing their homogeneous dispersion within the sintered bulk. In powder metallurgy processes, ball milling is commonly employed for effectively blending the additive and precursor powder. However, there are still several challenges associated with the ball milling process for blending purposes. For nanoparticles that are prone to form deformations, the collision of grinding balls may be responsible for generating deformations rather than achieving even distributions. Moreover, some powders may remain trapped at the corners of the jar during ball milling, making it difficult to achieve complete dispersion. Additionally, if nanoparticles have a tendency to react with the matrix material, excessive energy from ball milling can cause unintended reactions that prevent their incorporation as secondary phases. Alternatively, for chemical methods, the additive can be well dispersed in solution with the assistance of ultrasonic treatment or blended with purified precursor powders after isolation. However, complete removal of the organic solvent and reacted raw materials during separation and purification is challenging, potentially deteriorating the TE performance. The advantage of this ex situ nanocomposite strategy lies in its ability to effectively incorporate any desired nanoinclusion into the material; however, challenges concerning poor interface matching or potential interference during the sintering process may arise. Nevertheless, due to its clear purpose and intuitive effect, ex situ nanocomposite strategies have become a preference while designing bismuth telluride composites.

### 2.2 In Situ Nanocomposite Strategies

The in situ nanocomposite strategy refers to the direct precipitation of nanoinclusions at the grain boundaries of polycrystalline bulk materials. In comparison with the ex situ composite strategy, this approach offers improved interface quality between the introduced nanoinclusions and matrix due to the coherent growth of crystal lattices within the bulk material. Additionally, it ensures a more uniform dispersion of nanoinclusions throughout the matrix. However, controlling the composition of these precipitated nanoinclusions is quite challenging, as their formation is closely tied to process parameters during preparation. Consequently, designing experiments for synthesizing nanoinclusions with specific sizes and composition becomes difficult. Typically, researchers aim to realize elemental doping or alloying for targeted effects but unexpectedly discover that the generated nanoinclusions can enhance TE performance instead. Furthermore, achieving ideal results by directly adding these in situ‐introduced nanoinclusions through an ex situ composite strategy proves challenging due to interfacial bonding and unpredictable doping effects.

## 3 Mechanisms of TE Performance Enhancement through Nanocomposite Strategies

For both in situ and ex situ strategies to introduce nanoinclusions, the additive may react with matrix materials, thereby influencing their electrical or thermal transport properties. However, these variations are primarily due to the doping effect. The nanoinclusions themselves also play a crucial role in determining the electrical or thermal transport properties because electrons or phonons can transport within these nanoinclusions or across the resulting interfaces. The as‐observed, variable transport behaviors are highly dependent on the intrinsic transport properties of nanoinclusions, which are proportional to the volume fractions they occupy. Therefore, *κ*_L_ can be reduced by incorporating nanoinclusions with low *κ*_L_, while carrier mobility can be enhanced by incorporating nanoinclusions with high carrier mobility. However, the reality is not ideal as incorporated nanoinclusions usually have lower Seebeck coefficients than bismuth telluride, which may deteriorate the Seebeck coefficient in the resulting composites. Nevertheless, when their size reaches 1 nm, nanoscale effects may significantly alter the electrical properties of nanoinclusions and possibly enhance those of resultant nanocomposites. Additionally, due to the typical mean free paths of phonons and charge carriers being on tens of nanometers scale for bismuth telluride, the presence of nanoinclusion of such size can induce scattering effects on both phonons and charge carriers, leading to contrasting impacts on TE performance.^[^ ^25^, ^26^, ^27^, ^28^, ^29^ ^]^

Despite the nature of nanoinclusions themselves, the interfaces introduced by these nanoinclusions play a crucial role as well.^[^ ^30^ ^]^ It can even be said that such interface engineering primarily optimizes the TE performance. Two types of interfaces can be formed by nanoinclusions:^[^ ^31^ ^]^ 1) grain boundaries and 2) interfaces between the nanoinclusions and matrix materials. It is well established that nanoinclusions act as barriers to grain boundary movement, thereby impeding grain growth and increasing the density of grain boundaries. The presence of grain boundaries effectively scatters low‐frequency phonons, resulting in reduced *κ*_L_ at lower temperatures.^[^ ^32^ ^]^ For the interfaces between the nanoinclusions and matrix materials, although they inherently possess interfacial thermal resistance, this only becomes dominant when the size of nanoinclusions falls below the Kapitza radius. Meanwhile, fine nanoinclusions have a larger specific surface area, leading to a more pronounced interface effect. Additionally, due to the distinct crystalline structure of nanoinclusions, lattice mismatch may exist between the interfaces and matrix materials, yielding abundant point defects. These point defects primarily contribute to scattering high‐frequency phonons, consequently reducing *κ*_L_ in the low‐temperature range.^[^ ^33^, ^34^ ^]^ Moreover, these point defects may induce stronger scattering effects on charge carriers than acoustic phonon scattering, which will increase their scattering factor and hence the Seebeck coefficient.

However, these interfaces also act as scattering centers for charge carriers, impeding their transport.^[^ ^35^ ^]^ Despite the resulting decrease in charge carrier mobility, the overall electrical transport properties may not deteriorate due to the modified charge carrier scattering. Apart from the previously mentioned point defect, both the heterogeneous interfaces themselves and any impurities carried on them can contribute to charge carrier scattering. All of these additionally induced scattering effects can be referred to as “multiple scattering effects“, which increase the scattering factor and are conducive to enhancing the Seebeck coefficient. In addition, these interfaces selectively scatter charge carriers based on energy levels.^[^ ^36^ ^]^ The interface between bismuth telluride and the nanoinclusions can induce band structure bending that creates traps or barriers for charge carriers.^[^ ^37^ ^]^ The band bending is attributed to charge carrier transfer caused by the difference in work function. If metallic inclusions have a higher work function than the conduction band maximum of the matrix material, an energy barrier for electron transport is formed. Conversely, if the metallic inclusions have a lower work function than the valence band minimum of the matrix material, an energy barrier for hole transport is generated. Similarly, semiconducting nanoinclusions create energy barriers for electron or hole transport depending on the position of conduction or valence bands relative to those of the matrix materials. In cases where energy barriers are present, only charge carriers with higher energy than the barrier can participate in transport, leading to an increase in average carrier energy and consequently enhancing the Seebeck coefficient through a phenomenon known as energy filtering.^[^ ^22^ ^]^ The subsequent cases will elucidate these mechanisms more clearly.

## 4 Nanoinclusions for Nanocompositing Bismuth Telluride

After years of exploration, numerous nanoinclusions were found to effectively modulate the electrical and thermal transport properties of bismuth telluride. However, the preparation method and working mechanism for the nanocomposite of different nanoinclusions vary a lot, which can be classified based on the types of nanoinclusions.

### 4.1 Nonmetallic Hard Nanoparticle

The nonmetallic hard nanoparticle is a typical type of nanoinclusion that was initially employed in bismuth telluride due to its nonreactivity or limited reactivity with the material. Typically, these nanoparticles range in size from a few nanometers to tens of nanometers, and encompass various inorganic single substances (carbon (C),^[^ ^38^, ^39^ ^]^ silicon (Si),^[^ ^40^, ^41^ ^]^ etc.), covalent compounds (silicon carbide (SiC),^[^ ^42^, ^43^, ^44^, ^45^, ^46^, ^47^ ^]^ boron nitride (BN),^[^ ^48^, ^49^, ^50^, ^51^ ^]^ etc.), and metallic oxides (aluminum oxide (Al_2_O_3_),^[^ ^52^ ^]^ antimony oxides (Sb_2_O_3_),^[^ ^53^, ^54^, ^55^ ^]^ titanium dioxide (TiO_2_),^[^ ^56^ ^]^ etc.). **Table** **1** presents some representative cases of the incorporation of nonmetallic hard nanoparticles as nanoinclusions in bismuth telluride, which demonstrates their effectiveness in enhancing TE performance by adjusting the power factor (PF) and *κ*_L_. The incorporation of these nanoinclusions is typically achieved through an ex situ approach derived from powder processing techniques, such as ball milling, hand grinding, and so on. During the blending process, these nanoparticles exhibit high hardness and resistance against chemical reactions when subjected to high‐energy mechanical collisions. Hence, they are referred to as “hard nanoparticles” throughout this review. Consequently, blending them with bismuth telluride powders becomes a crucial step in fabricating the nanocomposite.

**Table 1:** Performance enhancement of bismuth telluride incorporated with some different nonmetallic hard nanoparticles. (The data have been extracted from the figures, and may differ from the textual descriptions provided by the authors).

Type	Matrix	Nanoinclusion	Size [nm]	Increase in PF [%]	Decrease in *κ*_L_ [%]	Peak *ZT* before nanocomposite	Peak *ZT* after nanocomposite	Increase in peak *ZT* [%]	Year and references
P	Bi_0.3_Sb_1.7_Te_3_	SiC	100	18.3	5.2	1.23 at 423 K	1.33 at 373 K	8.5	2013^[^ ^42^ ^]^
P	Bi_0.4_Sb_1.6_Te_3_	*a*‐SiO_2_	50	15.7	13.6	1.22 at 412 K	1.27 at 363 K	3.7	2013^[^ ^65^ ^]^
P	Bi_0.4_Sb_1.6_Te_3_–3 wt% Te	*a*‐Si_3_N_4_	25	−17.0	32.9	1.29 at 383 K	1.38 at 383 K	7.1	2015^[^ ^66^ ^]^
P	Bi_0.4_Sb_1.6_Te_3_	*β*‐Zn_4_Sb_3_	40–250	−38.9	6.1	1.21 at 403 K	1.43 at 443 K	18.2	2016^[^ ^68^ ^]^
P	Bi_0.5_Sb_1.5_Te_3_	Ta_2_O_5_	100–150	5.2	−29.7	1.08 at 300 K	1.38 at 300 K	27.0	2017^[^ ^67^ ^]^
P	Bi_0.5_Sb_1.5_Te_3_	Y_2_O_3_	30–70	9.1	−31.6	1.12 at 350 K	1.24 at 300 K	10.3	2017^[^ ^70^ ^]^
P	Bi_0.5_Sb_1.5_Te_3_	Sb_2_O_3_	<200	13.8	−9.0	1.22 at 325 K	1.51 at 350 K	24.3	2018^[^ ^53^ ^]^
P	Bi_0.5_Sb_1.5_Te_3_	C	5	38.9	30.4	0.78 at 349 K	1.25 at 323 K	60.3	2019^[^ ^38^ ^]^
P	Bi_0.5_Sb_1.5_Te_3_	Fe_3_O_4_	9–18	2.2	23.4	1.15 at 338 K	1.50 at 338 K	30.5	2020^[^ ^72^ ^]^
N	Bi_2_Te_2.7_Se_0.3_	*γ*‐Al_2_O_3_	20	31.2	27.0	0.73 at 448 K	0.99 at 405 K	35.6	2011^[^ ^63^ ^]^
N	Bi_2_Te_2.7_Se_0.3_	Y_2_O_3_	10–30	16.2	51.5	0.75 at 425 K	1.21 at 367 K	62.1	2021^[^ ^71^ ^]^
N	Bi_2_Te_2.7_Se_0.3_	TiO_2_	–	55.7	27.1	0.86 at 387 K	1.31 at 365 K	52.7	2023^[^ ^56^ ^]^
N	Bi_2_Te_2.7_Se_0.3_	Fe_3_O_4_	200	13.0	14.9	0.62 at 422 K	0.72 at 397 K	15.6	2023^[^ ^111^ ^]^

**Figure 2:**
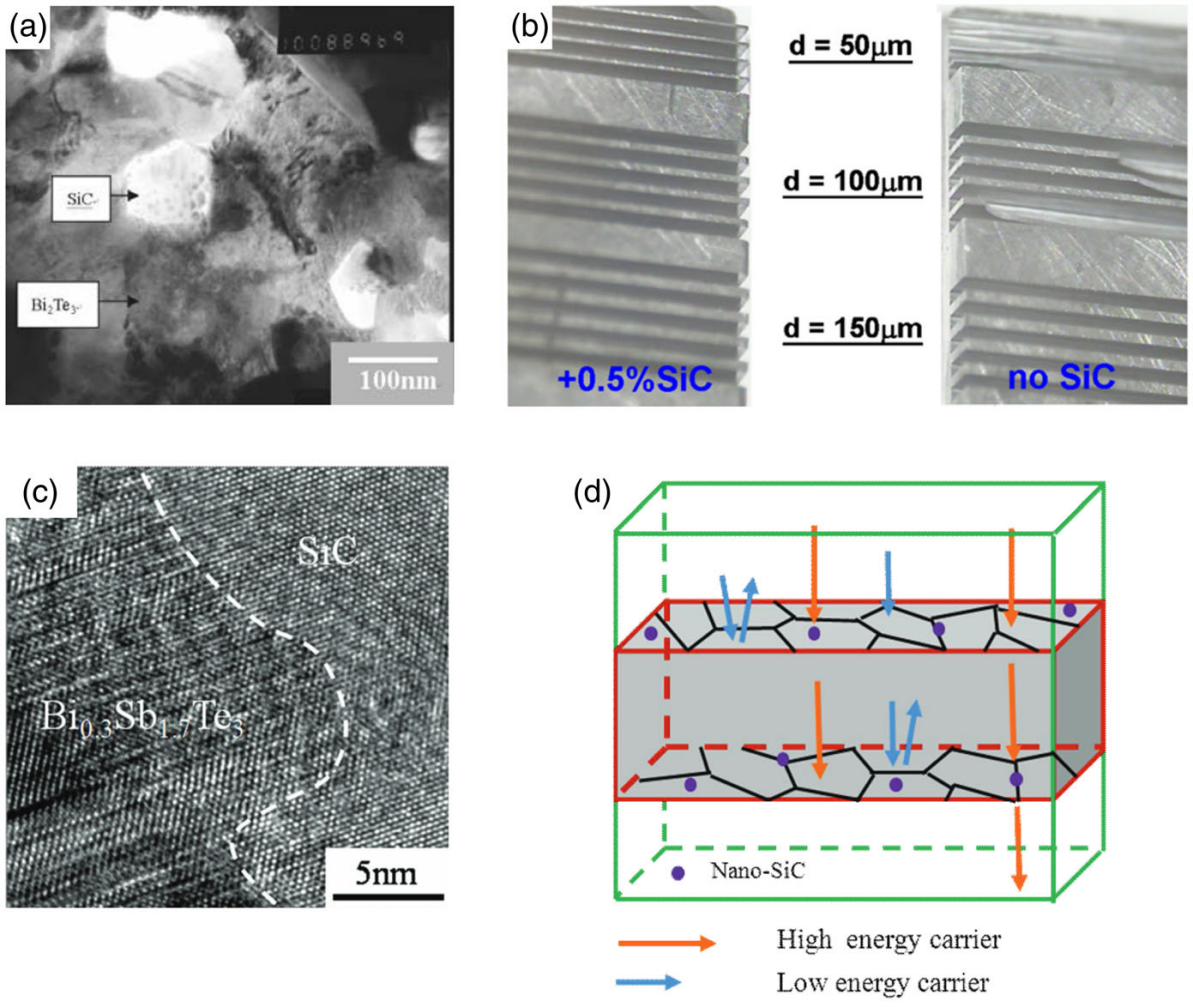
a) TEM image of the Bi_2_Te_3_ sample incorporated with 0.5 wt% SiC. Reproduced with permission.^[^ ^43^ ^]^ Copyright 2006, WILEY‐VCH Verlag GmbH & Co. KGaA, Weinheim. b) The morphologies of microslabs with different thicknesses made on the Bi_0.5_Sb_1.5_Te_3_ without and with 0.5% SiC in volume. Reproduced with permission.^[^ ^109^ ^]^ Copyright 2006, IOP Publishing Ltd. c) High‐resolution TEM (HRTEM) image of the interface between Bi_0.3_Sb_1.7_Te_3_ matrix and SiC.^[^ ^42^ ^]^ d) Schematic diagram of the energy‐filtering effect induced by SiC nanoparticles. Reproduced with permission.^[^ ^42^ ^]^ Copyright 2013, WILEY‐VCH Verlag GmbH & Co. KGaA, Weinheim.

The incorporation of nonmetallic hard particles was initially attempted in some TE materials with high *κ*_L_, such as Si–Ge and Bi–Sb alloys, to enhance phonon scattering. The BN, ZrO_2_, and Si_3_N_4_ particles were first used in these TE materials and successfully resulted in a 10–40% improvement in TE performance.^[^ ^57^, ^58^, ^59^, ^60^, ^61^ ^]^ Despite not necessarily selecting particles at the nanoscale, these incorporated particles exhibited a positive effect. These findings inspired researchers to explore this strategy further in bismuth telluride. In 1993, Fleurial predicted that dispersing fine inclusions within p‐type Bi_2_Te_3_‐based alloys could potentially improve the *ZT* value by 15–20% at 200–300 K, which has guided subsequent research efforts.^[^ ^62^ ^]^ As BN is widely used for incorporation, it naturally became the focus of initial attempts on bismuth telluride. However, it was discovered that incorporating BN (≈5–10 μm) actually deteriorated the *ZT* value due to increased electrical resistivity.^[^ ^48^, ^49^, ^50^ ^]^ Similar phenomena were also observed when incorporating other particles like MgO.^[^ ^62^ ^]^ Therefore, a key issue for incorporating hard particles into bismuth telluride is to avoid the deterioration of electrical transport properties. For this purpose, our group selected SiC nanoparticles for incorporation,^[^ ^42^, ^43^, ^45^ ^]^ which are also promising TE semiconductors with higher electrical conductance compared to BN. On the other hand, these SiC nanoparticles have an average size of about 100 nm, which may be conducive to phonon scattering. As anticipated, our work resulted in some improvement in the electrical transport properties. However, unexpectedly, this enhancement was attributed to an increase in the Seebeck coefficient. In 2008, we achieved a *ZT* value of 1.04 for Bi_2_Te_3_ by incorporating 0.1 vol% SiC nanoparticles, which can be observed embedded at the grain boundaries (**Figure** **2**a). It was speculated that the introduction of oxygen caused the increased Seebeck coefficient at that time; however, no clear explanation was provided at that time. Additionally, we found that incorporating SiC nanoparticles strengthened bismuth telluride and enabled it to be diced into walls as thin as 50 μm (Figure 2b). The success achieved with SiC nanoparticles prompted further research on identifying nanoparticles capable of enhancing electrical transport properties.

In 2011, Li et al. discovered that the incorporation of *γ*‐Al_2_O_3_ into Bi_2_Se_0.3_Te_2.7_ not only reduced the thermal conductivity but also effectively enhanced the electrical transport properties by increasing the Seebeck coefficient.^[^ ^63^ ^]^ The incorporation of *γ*‐Al_2_O_3_ can lead to a 35% increase in the peak *ZT* value compared to the sample without *γ*‐Al_2_O_3_. They attributed this phenomenon to potential barrier scattering, suggesting that the introduction of *γ*‐Al_2_O_3_ nanoparticles introduced imperfect structures (point defects and dislocations) at grain boundaries, resulting in localized states that enhance potential barrier height. As a degenerate semiconductor, the enhanced potential barrier height leads to a higher scattering factor, thereby increasing the Seebeck coefficient. In 2013, our group further investigated the effect of incorporating SiC nanoparticles with an average size of about 100 nm into Bi_0.3_Sb_1.7_Te_3_.^[^ ^42^ ^]^ The interfaces between SiC nanoparticles and the matrix were found to be coherent interfaces (Figure 2c). By introducing 0.4 vol% SiC nanoparticles, significant enhancement in the Seebeck coefficient was observed, resulting in an increase in the *ZT* value from 1.23 to 1.33. The energy‐filtering effect was introduced in this study based on Dresselhaus’ theory,^[^ ^64^ ^]^ for which the schematic diagram is illustrated in Figure 2d. Since then, more and more researchers have believed that more nanoinclusions can produce this potential barrier scattering or energy‐filtering effect. The physical and chemical properties of nonmetallic hard nanoparticles are stable. By the ex situ incorporation strategy, nanoinclusions with a similar energy‐filtering effect can be screened more quickly. A large number of nanoinclusions that can improve the TE performance of bismuth telluride have also been reported, and the TE performance of bismuth telluride has been greatly improved.

Shortly after the publication of our group's work, Dou et al. reported the energy‐filtering effect of *a*‐SiO_2_ with a particle size of 50 nm in bismuth telluride.^[^ ^65^ ^]^ By incorporating 0.55 vol% *a*‐SiO_2_ nanoparticles, they observed an increase in the scattering factor from −0.5 to −0.35, demonstrating the characteristic energy‐filtering effect. Meanwhile, *κ*_L_ decreased monotonically with increasing *a*‐SiO_2_ content at a temperature lower than 320 K, which can be attributed to enhanced phonon scattering by the embedded nanoparticles and the phase boundaries. The peak *ZT* value of Bi_0.4_Sb_1.6_Te_3_ can be increased from 1.22 to 1.27 with the incorporation of 0.55 vol% *a*‐SiO_2_ nanoparticles. Then in 2015, Dou et al. found that amorphous Si_3_N_4_ (*a*‐Si_3_N_4_) nanoparticles (≈25 nm) exhibited a similar effect, increasing the *ZT* value to 1.38.^[^ ^66^ ^]^ Therefore, one may speculate that the nanoparticles of silicon compounds may have similar effects, but this has not been confirmed so far and needs to be further explored.

In fact, the energy‐filtering effect is primarily determined by the energy band structure of the nanoparticle. When nonmetallic hard nanoparticles form a potential barrier at the interface with bismuth telluride, they can induce an energy‐filtering effect on low‐energy carriers. For p‐ and n‐type bismuth telluride, it is essential to establish potential barriers at the valence and conduction bands, respectively. This property has been observed in various types of nonmetallic hard nanoparticles over the past decade, including Ta_2_O_5_,^[^ ^67^ ^]^ Zn_4_Sb_3_,^[^ ^68^ ^]^ ZnO,^[^ ^69^ ^]^ Y_2_O_3_,^[^ ^70^ ^]^ Sb_2_O_3_,^[^ ^53^, ^55^ ^]^ and so on for p‐type bismuth telluride, as well as Al_2_O_3_,^[^ ^63^ ^]^ TiO_2_,^[^ ^56^ ^]^ Y_2_O_3_,^[^ ^71^ ^]^ and so on for n‐type bismuth telluride. The incorporation of these nonmetallic hard nanoparticles can significantly enhance the TE performance of bismuth telluride. Among them, Sb_2_O_3_ exhibits the most pronounced effect on p‐type bismuth telluride. The incorporation of 4 wt% Sb_2_O_3_ with particle size <200 nm can increase the peak *ZT* by 26% to 1.51 at 350 K.^[^ ^53^ ^]^ Similarly, TiO_2_ shows remarkable effects on n‐type bismuth telluride as the addition of 3 wt% TiO_2_ can increase peak *ZT* by 55% to 1.31 at 373 K.^[^ ^56^ ^]^

However, the current supporting evidence for the energy‐filtering effect of these nonmetallic hard nanoparticles is insufficient. It is not methodologically rigorous to rely solely on the single parabolic band (SPB) model for assessing value change. As previously mentioned, both the imperfect structure at the interface and the semiconductor contact itself contribute to interface barriers. The specific mechanism responsible for generating interface barriers in materials that exhibit an energy‐filtering effect remains unconfirmed.

**Figure 3:**
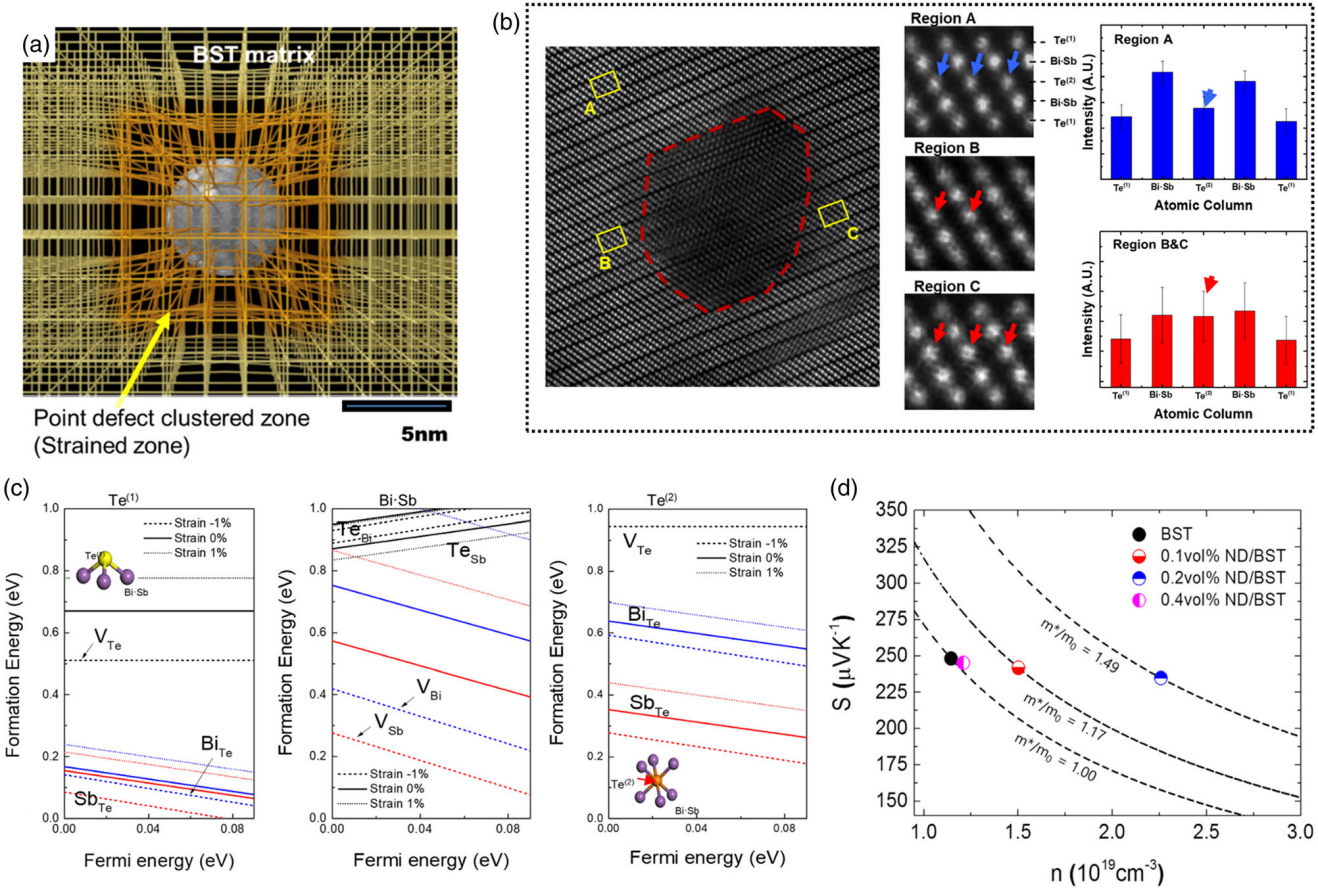
a) Schematic diagram of the point defect clustered zone induced by the strain due to the nanodiamonds. b) HAADF–STEM images according to the $\left[01\bar{1}1\right]$ zone axis as well as the comparison of the intensity levels of each atom to confirm the atomic configuration in the A region and in the B and C regions. c) Point‐defect formation energy in Te(1), Bi·Sb, and Te(2) atoms of cation‐rich Bi_0.5_Sb_1.5_Te_3_ with different strains obtained from a DFT calculation of the formation of intrinsic point defects in BST materials with strains. d) Theoretical relationship between Seebeck coefficient and carrier concentration based on the effective mass of the carriers. Reproduced with permission.^[^ ^38^ ^]^ Copyright 2018, Elsevier Ltd.

Nevertheless, for some nonconductive insulators, it can be determined that the energy‐filtering effect is caused by the imperfect structure at the interface. Kim et al. incorporated nanodiamonds with size of ≈5 nm into Bi_0.5_Sb_1.5_Te_3_.^[^ ^38^ ^]^ They attributed the improved TE performance to the clustering of point defects (e.g., V_Sb_, V_Bi_, Sb_Te_, Bi_Te_, and V_Te_) cluster at the interface within a few nm range originating from nanodiamonds, as schematically demonstrated in **Figure** **3**a. High‐angle annular dark field (HAADF)–scanning transmission electron microscopy (STEM) analysis reveals higher Te(1)/Bi,Sb atom‐intensity ratios but lower Te(1)/Te(2) atom‐intensity ratios near the interface compared to regions away from it, indicating an increasing number of vacancies and antisite defects near the interface (Figure 3b). Density functional theory (DFT) results suggest that these defects exhibit significantly reduced formation energy under tensile strain induced by the nanodiamonds (Figure 3c); the formation energy of these defects decreases significantly. This metastable interface exhibits significant residual strain and effectively hosts these point defects. As shown by the Pisarenko curve, the points of samples with the incorporation of nanodiamond deviate upward from the curve, indicating increased density‐of‐state effective mass or scattering factor (Figure 3d). Although the author did not mention the energy‐filtering effect, the electrical transport properties show typical characteristics of the energy‐filtering effect. More importantly, they revealed that these point defects generated by the interface have a strong scattering effect on phonons, which can significantly reduce the *κ*_L_.

These generated point defects constitute a significant portion of the interfacial defects. In fact, due to the limited reactivity of nonmetallic hard nanoparticles with the matrix material, the interfacial defects they introduce may exert a greater influence on the TE performance of bismuth telluride than nanoinclusions. The interfacial defects induced by the imperfect structure at the interface are highly sensitive to the fabrication process. Consequently, different processes used for preparing bismuth telluride‐based nanocomposites may yield various outcomes. For instance, recent findings by Chen et al. demonstrated that BN particles, previously reported to deteriorate the TE performance of bismuth telluride, can actually enhance the *ZT* value from 1.0 to 1.3 through energy‐filtering effects and enhanced phonon scattering mechanisms.^[^ ^51^ ^]^ A notable distinction between Chen et al.'s study and earlier reports lies in their utilization of a rocking melting method instead of mechanical alloying for precursor preparation.^[^ ^51^ ^]^ This suggests that rocking melting could potentially induce more interfacial defects, thereby leading to energy‐filtering effects and enhanced phonon scattering.

**Figure 4:**
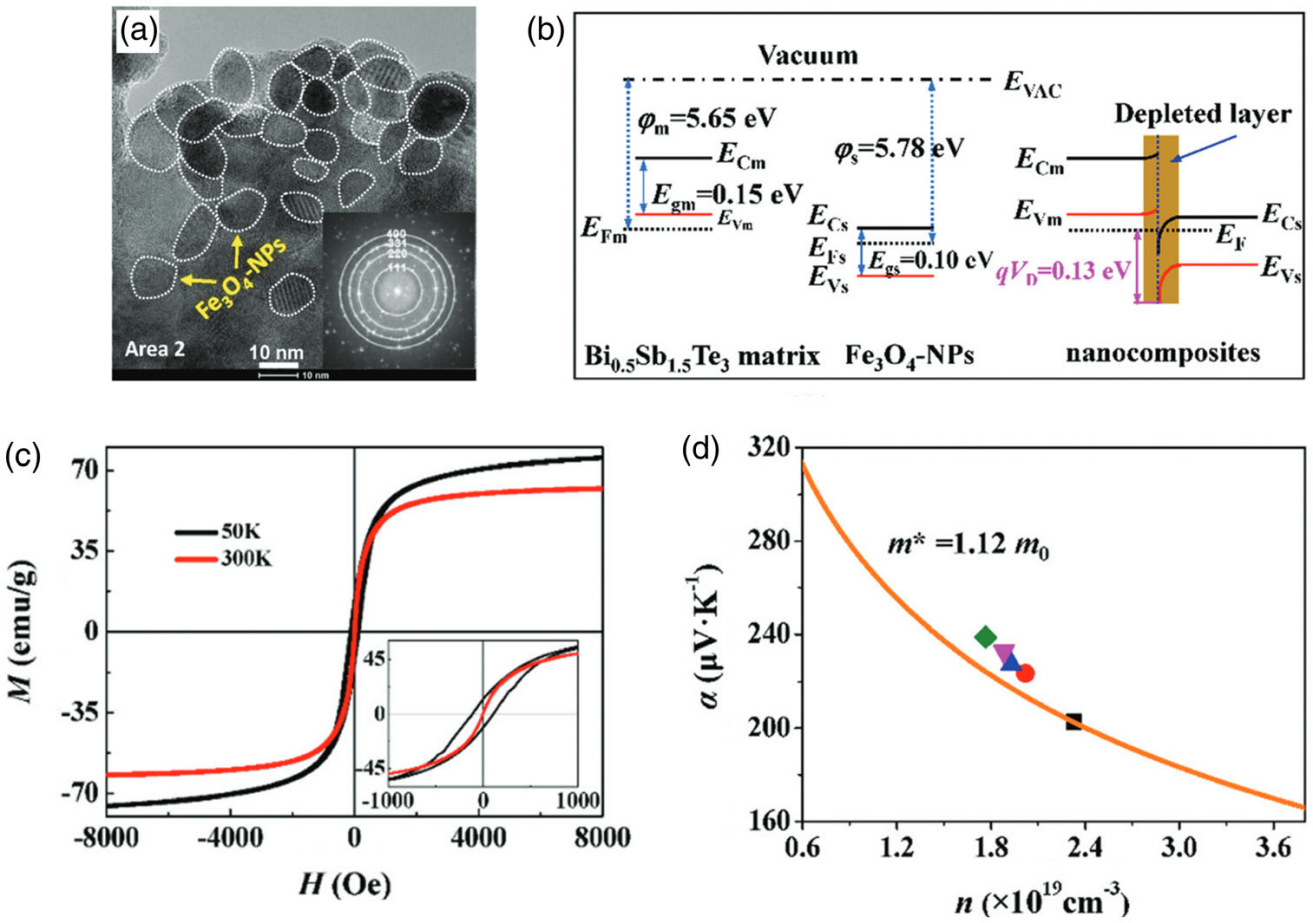
a) HRTEM image of Bi_0.5_Sb_1.5_Te_3_ incorporated with 0.15 wt% Fe_3_O_4_ nanoparticles (the inset is the corresponding FFT pattern). b) *M*–*H* curves of Fe_3_O_4_ nanoparticles at 50 and 300 K. c) The diagram of the electronic structure near the interface between the Fe_3_O_4_ nanoparticles and the Bi_0.5_Sb_1.5_Te_3_ matrix. d) Seebeck coefficient versus carrier concentration curve of the Bi_0.5_Sb_1.5_Te_3_ matrix at 300 K obtained by the Pisarenko relation using m* = 1.12 *m*_0_. Reproduced with permission.^[^ ^72^ ^]^ Copyright 2020, The Royal Society of Chemistry.

In addition, other effects are documented to potentially influence interface scattering, such as the magnetic properties of nanoparticles. In 2020, Li et al. demonstrated the incorporation of Fe_3_O_4_ magnetic nanoparticles with an average particle size ranging from 9 to 18 nm into Bi_0.5_Sb_1.5_Te_3_ materials (**Figure** **4**a).^[^ ^72^ ^]^ However, the height of the Schottky potential barrier formed by the contact between Fe_3_O_4_ and the matrix material is just ≈0.13 eV (Figure 4b), which is insufficient to induce the energy‐filtering effect. Nevertheless, within the working temperature range (300–500 K), these Fe_3_O_4_ nanoparticles exhibit a superparamagnetic state (Figure 4c) where thermal fluctuations randomly alter their magnetic moments. This leads to enhanced carrier multiple scattering effect through interactions between magnetic moments and electron spins. Therefore, incorporating Fe_3_O_4_ nanoparticles can result in an increase in the scattering parameter (Figure 4d), boosting the Seebeck coefficient while maintaining a high PF even when the electrical conductivity is at reduced levels. The notable reduction in thermal conductivity can be attributed to amplified phonon scattering arising from fluctuations in magnetic moments and newly formed heterointerfaces. Notably, the nanocomposite containing 0.15 wt% Fe_3_O_4_ nanoparticles exhibited an outstanding *ZT* value of 1.5 at 340 K.

Although the specific mechanism remains incompletely understood, it is evident that the interface between these nonmetallic hard nanoparticles and bismuth telluride exhibits a distinctive scattering effect on charge carriers. The origin of this scattering phenomenon may be attributed to either the fabrication process or the unique properties inherent in these nanoparticles, necessitating further investigations.

### 4.2 Metallic Nanoparticles

In addition to the aforementioned nonmetallic hard nanoparticles, metallic nanoparticles are also commonly utilized for incorporating bismuth telluride. **Table** **2** presents some favorable outcomes of integrating metallic nanoparticles into bismuth telluride. It is worth noting that the trends of PF and *κ*_L_ significantly vary with the incorporation of different metallic particles, which may be attributed to the inevitable doping effect of the metallic nanoparticles.

**Table 2:** Performance enhancement of bismuth telluride incorporated with some different metallic nanoparticles. (The data have been extracted from the figures, and may differ from the textual descriptions provided by the authors).

Type	Matrix	Nanoinclusion	Size	Increase in PF [%]	Decrease in *κ*_L_ [%]	Peak *ZT* before nanocomposite	Peak *ZT* after nanocomposite	Increase in peak *ZT* [%]	Year and references
P	Bi_0.4_Sb_1.6_Te_3_	Au	10–100 nm	−6.5	15.0	0.86 at 373 K	1.00 at 424 K	16.7	2014^[^ ^74^ ^]^
P	Bi_0.5_Sb_1.5_Te_3_	Co	–	14.6	21.6	0.98 at 382 K	1.10 at 367 K	12.4	2020^[^ ^77^ ^]^
P	Bi_0.5_Sb_1.5_Te_3_	Fe	–	0.9	37.4	0.98 at 376 K	1.17 at 369 K	19.5	2020^[^ ^77^ ^]^
N	Bi_2_Te_3_	Ag	60 nm	−49.7	61.7	0.23 at 324 K	0.77 at 475 K	230.5	2015^[^ ^73^ ^]^
N	Bi_2_Te_3_	Au	10 nm	1.8	−12.9	0.56 at 300 K	0.95 at 480 K	69.6	2016^[^ ^75^ ^]^
N	Bi_2_Te_2.7_Se_0.3_	Ni	10–20 nm	67.2	0.0	0.46 at 449 K	0.66 at 425 K	43.5	2019^[^ ^84^ ^]^
N	Bi_2_Te_2.7_Se_0.3_	Ni	8–10 nm	17.8	−2.5	0.81 at 369 K	1.08 at 359 K	32.4	2020^[^ ^76^ ^]^
N	Bi_2_Te_2.8_Se_0.2_	Au	<500 μm	66.1	7.2	0.79 at 475 K	1.05 at 375 K	32.4	2021^[^ ^81^ ^]^

Compared with nonmetallic hard nanoparticles, one advantage of incorporating metallic nanoparticles is their obtainability through reduction reactions using metal source in solution form, such as silver nitrate (AgNO_3_),^[^ ^73^ ^]^ gold chloride hydrate (HAuCl_4_),^[^ ^74^, ^75^ ^]^ nickel(II) acetylacetonate (Ni(acac)_2_),^[^ ^76^, ^77^ ^]^ and so on. This facilitates the refinement of metallic particles and ensures the homogeneous dispersion of nanoparticles throughout the bulk material. For instance, by dispersing Sb microparticles into an aqueous solution containing HAuCl_4_, the subsequent reaction yields Au nanoparticles supported on Sb microparticles, which can serve as a precursor for facile synthesis of bulk materials incorporated with Au nanoparticles.^[^ ^74^ ^]^ Additionally, Bi_2_Te_3_ nanopowders and Ag nanoparticles can be separately synthesized using a hydrothermal method. The synthesized Ag nanoparticles can then be uniformly mixed with Bi_2_Te_3_ powders in absolute ethyl alcohol through ultrasound and stirring, resulting in the formation of a Bi_2_Te_3_‐based nanocomposite with a uniform distribution of Ag nanoparticles.^[^ ^73^ ^]^ However, some researchers have also successfully introduced metallic nanoparticles by utilizing micrometer‐scale metal particles as initial raw materials.^[^ ^78^, ^79^ ^]^ This phenomenon may be attributed to the reaction between metal particles and bismuth telluride, resulting in residual nanoscale particles. The incorporation of metallic nanoparticles into bismuth telluride poses a challenge as it can potentially alter carrier concentration and energy band structures due to doping effects.

In addition to the doping effect, the incorporated metallic nanoparticles in bismuth telluride play a role similar to the nonmetallic hard nanoparticles. The metallic nanoparticles can also realize the energy‐filtering effect, carrier multiple scattering effect, and phonon scattering effect, thereby resulting in improvement in TE performance. However, unlike contacts between semiconductors, the interface between metal and bismuth telluride involves a distinct metal–semiconductor contact. When they are forming the junction, electrons will transfer from the metal to bismuth telluride, thereby changing the charge carrier concentration. The transfer of electrons depends on the work function of the metal, which varies depending on the crystal orientations and surface impurities. Besides, the bending of the band structure of bismuth telluride at this interface is also attributed to the work function of the metal employed, which determines whether a barrier or a well is formed at the interface. Therefore, the proper work function of the metallic nanoparticle is required to induce an energy‐filtering effect and properly adjust the carrier concentration for the bismuth telluride. Based on theoretical calculations regarding the enhancement of PF, the potential barrier of ≈0.1 eV would be most suitable.^[^ ^80^ ^]^ Considering the work function range of metals, it is obvious that the successful cases of energy‐filtering effect achieved by metallic nanoparticle composite will be more concentrated in n‐type bismuth telluride.

**Figure 5:**
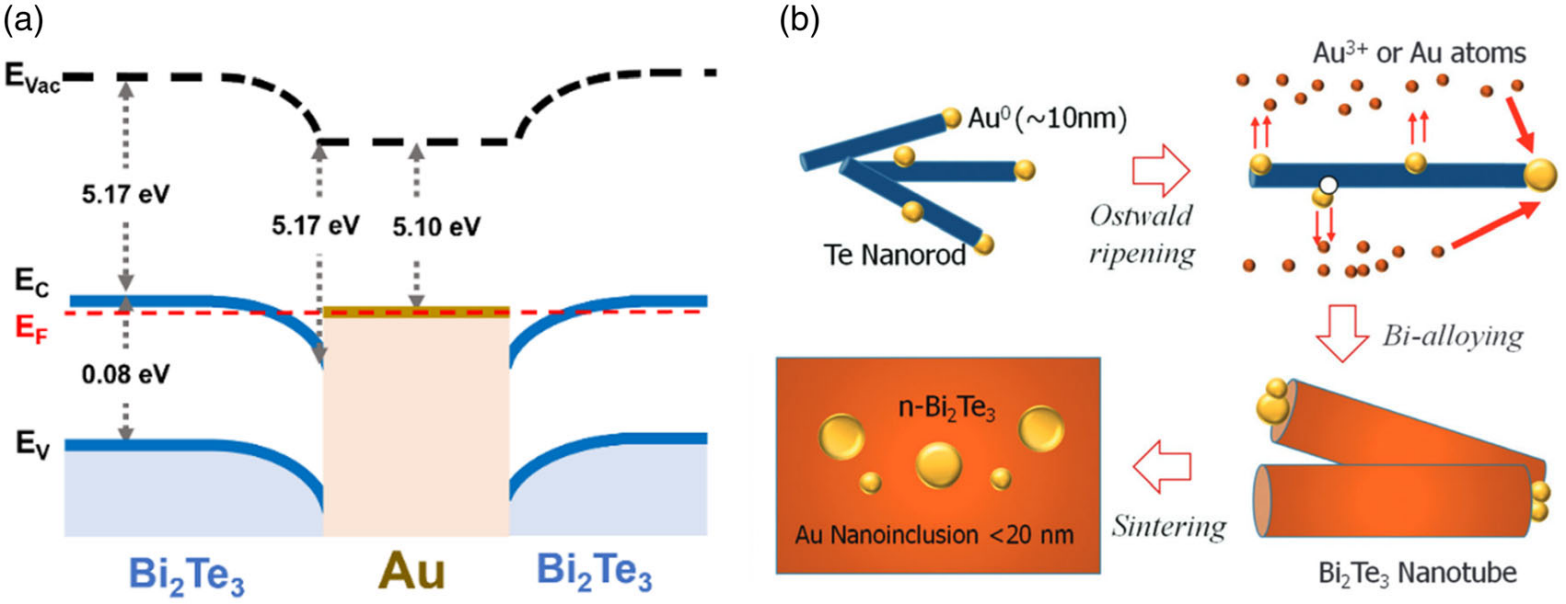
a) Schematic diagram of electronic structure between the Bi_2_Te_3_ and Au. Reproduced with permission.^[^ ^75^ ^]^ Copyright 2021, American Chemical Society. b) Schematic diagram of the hybrid synthesis procedure of Au‐nanodot/Bi_2_Te_3_ nanotube. Reproduced with permission.^[^ ^81^ ^]^ Copyright 2016, The Royal Society of Chemistry.

In comparison with other metals, Au exhibits a work function of ≈5.1 eV, enabling the formation of a potential barrier (≈0.1 eV) at its interface with n‐type bismuth telluride, as schematically demonstrated in **Figure** **5**a. Lee et al. successfully synthesized Au nanodot‐embedded Bi_2_Te_3_ composites with enhanced TE performance.^[^ ^75^ ^]^ In their work, Au nanodots were first utilized as seeds for the growth of Te nanorods, followed by the diffusion of Bi into the Te nanorods to form Bi_2_Te_3_ nanotubes containing Au nanodots. Sintering these nanotubes effectively yielded n‐type Bi_2_Te_3_ incorporated with Au nanoinclusions smaller than 20 nm. The schematic diagram of the synthesis procedure is illustrated in Figure 5b. The incorporation of a small amount of Au nanodots effectively increased the PF due to the energy‐filtering effect and reduced *κ*_L_ through intensified phonon scattering at the Au–Bi_2_Te_3_ interface. A maximum *ZT* value of 0.95 was obtained at 480 K in 2.0 mol% Au nanodot embedded Bi_2_Te_3_, which was ≈67% higher than that of pristine Bi_2_Te_3_. Kawajiri et al. synthesized the nanocomposite by simply mixing Bi_2_Te_3−*x*_Se_*x*_ powders with spherical gold microparticles.^[^ ^81^ ^]^ Through this approach, Au nanoparticles were successfully incorporated into the bulk material, accompanied by the formation of AuTe_2_. As previously mentioned, this phenomenon can be attributed to a reaction between Au and Bi_2_Te_3−*x*_Se_*x*_, resulting in a size reduction of the Au particles. Additionally, these findings validate the energy‐filtering effect induced by incorporated Au in Bi_2_Te_2.8_Se_0.2_, leading to a similar enhancement in PF. Together with reduced *κ*_L_, an improved *ZT* value of 1.0 was achieved at room temperature.

In addition to the band bending induced by metal–semiconductor contact, the magnetic‐drag effects and spin entropy for certain magnetic metallic nanoparticles can also enhance the TE performance. This phenomenon was initially observed in Ba_0.3_In_0.3_Co_4_Sb_12_ doped with Co nanoparticles in 2017 and subsequently identified in various other TE materials, including bismuth telluride.^[^ ^23^ ^]^ It is worth noting that this phenomenon only occurs when the particle size is reduced to a specific value.^[^ ^82^ ^]^ When the size of a ferromagnetic nanoparticle is small enough to contain one magnetic domain, it can be magnetized into a paramagnet under an external magnetic field, which is referred to as “superparamagnetism”.^[^ ^83^ ^]^ At this stage, electrons are transferred from the paramagnetic nanoparticles to the matrix materials, altering the charge carrier concentration similar to nonmagnetic metallic nanoparticles. Additionally, the transition of nanoparticles from ferromagnetism to superparamagnetism induces magnetic fluctuations that cause multiple scattering of electrons, particularly those with lower energy levels; thus, this results in an energy‐filtering effect.

**Figure 6:**
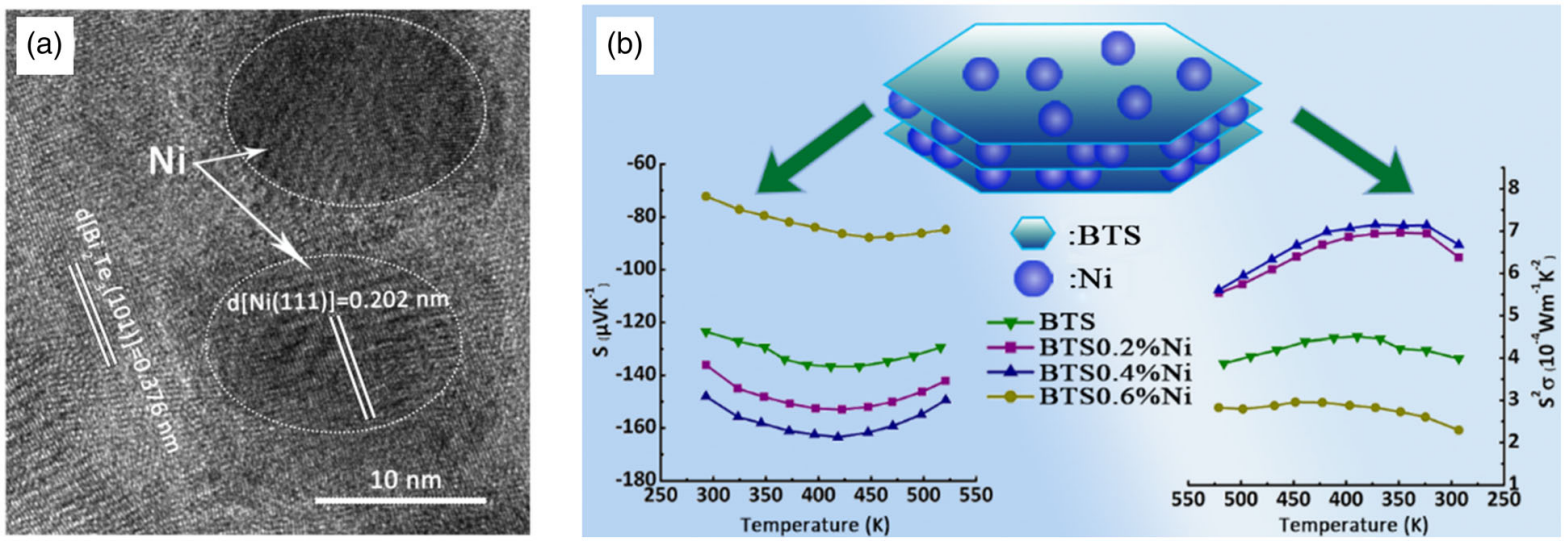
a) HRTEM image of Bi_2_Te_2.7_Se_0.3_ incorporated with 0.4 mol% Ni nanoparticles. b) Schematic diagram of Bi_2_Te_2.7_Se_0.3_/Ni nanocomposite and the temperature dependence of Seebeck coefficient and PF for the Bi_2_Te_2.7_Se_0.3_/Ni nanocomposite with different amounts of Ni. Reproduced with permission.^[^ ^84^ ^]^ Copyright 2019, American Chemical Society.

Du et al. investigated the incorporation of Ni nanoparticles into n‐type Bi_2_Te_2.7_Se_0.3_, confirming that the Ni nanoparticles underwent a magnetic transition from ferromagnetism to superparamagnetism at ≈315 K.^[^ ^84^ ^]^ The inclusion of Ni <20 nm can be detected in the Bi_2_Te_2.7_Se_0.3_ incorporated with 0.4 vol% Ni nanoparticles (**Figure** **6**a), which resulted in reduced remnant magnetization and coercivity for Bi_2_Te_2.7_Se_0.3_ at 350 K, leading to a significant enhancement in the carrier scattering factor that enhanced Seebeck effect (Figure 6b). Additionally, the metal–semiconductor contact increased carrier concentration. As a result of increased PF and decreased *κ*_L_, the peak *ZT* value exhibited a ≈43% increase to reach 0.66 at 425 K, demonstrating the significant impact of interface engineering through Ni nanoinclusions. Subsequently, Ma et al. discovered a new preparation process using low‐temperature and high‐pressure sintering methods that can suppress chemical reactions between Ni and Bi_2_Te_2.7_Se_0.3_ at the interface while ensuring the incorporation of superparamagnetic Ni nanoparticles into the Bi_2_Te_2.7_Se_0.3_ matrix.^[^ ^76^ ^]^ The mechanism for improvement is similar to regular preparation processes with higher temperature and lower pressure; however, in this case, the *ZT* value can be further improved to ≈1.1. Therefore, suppressing the reaction between magnetic metallic particles and Bi_2_Te_2.7_ Se_0.3_ matrix may play a crucial role in achieving high *ZT* values.

However, reactions with certain other magnetic metals could also improve TE performance. Wei et al. investigated the incorporation of magnetic Fe/Co into p‐type Bi_0.48_Sb_1.52_Te_3_ by adding single substances as raw materials. Although it is difficult for magnetic Fe and Co to enter the lattice of bismuth telluride due to the “like dissolves like” rule, parts of Fe and Co can react with the matrix and result in a doping effect.^[^ ^77^ ^]^ Meanwhile, a limited effect of the ferro‐superparamagnetic transition was observed, which may be attributed to the insufficient reduction in the size of the Fe/Co powders. Only a certain degree of ferromagnetism can be observed in the Fe/Co‐doped sample. However, the doping effect led to increases in density‐of‐state effective mass, bandgap, and carrier concentration, along with a decrease in *κ*_L_, achieving a peak *ZT* value of ≈1.15.

Therefore, the key to incorporating metallic nanoparticles into bismuth telluride lies in regulating the reaction between metallic nanoparticles and bismuth telluride. This involves controlling the size of metal nanoparticles to ensure effective phonon scattering, as well as facilitating the transition from a ferromagnetic to a superparamagnetic phase for magnetic metals. The reaction also determines the extent of metal doping within the matrix, which influences carrier concentration and band structures. Furthermore, it significantly impacts the interface between metallic nanoparticles and the bismuth telluride matrix, which plays a pivotal role in minimizing carrier scattering while enhancing phonon scattering.

### 4.3 Compounds with Low Thermal Conductivity

To address the challenges associated with incorporating metal nanoparticles, an alternative approach can be proposed by considering the incorporation of compounds with low thermal conductivity. Unlike metals that possess high thermal conductivity, these compounds themselves contribute to reducing the overall thermal conductivity through a composite effect. Moreover, copper or silver‐based tellurides or selenides are commonly used as compounds with low thermal conductivity. Numerous studies have demonstrated that adding a certain amount of copper or silver ions into both n‐ and p‐type bismuth telluride matrices can enhance their TE performance. Therefore, even if doping reactions occur, it is possible to optimize the TE performance to some extent. However, it should be noted that incorporating compounds with low thermal conductivity does not necessarily result in the formation of nanoinclusions. This section solely presents a strategy for improving TE performance from the perspective of nanocomposites.

**Figure 7:**
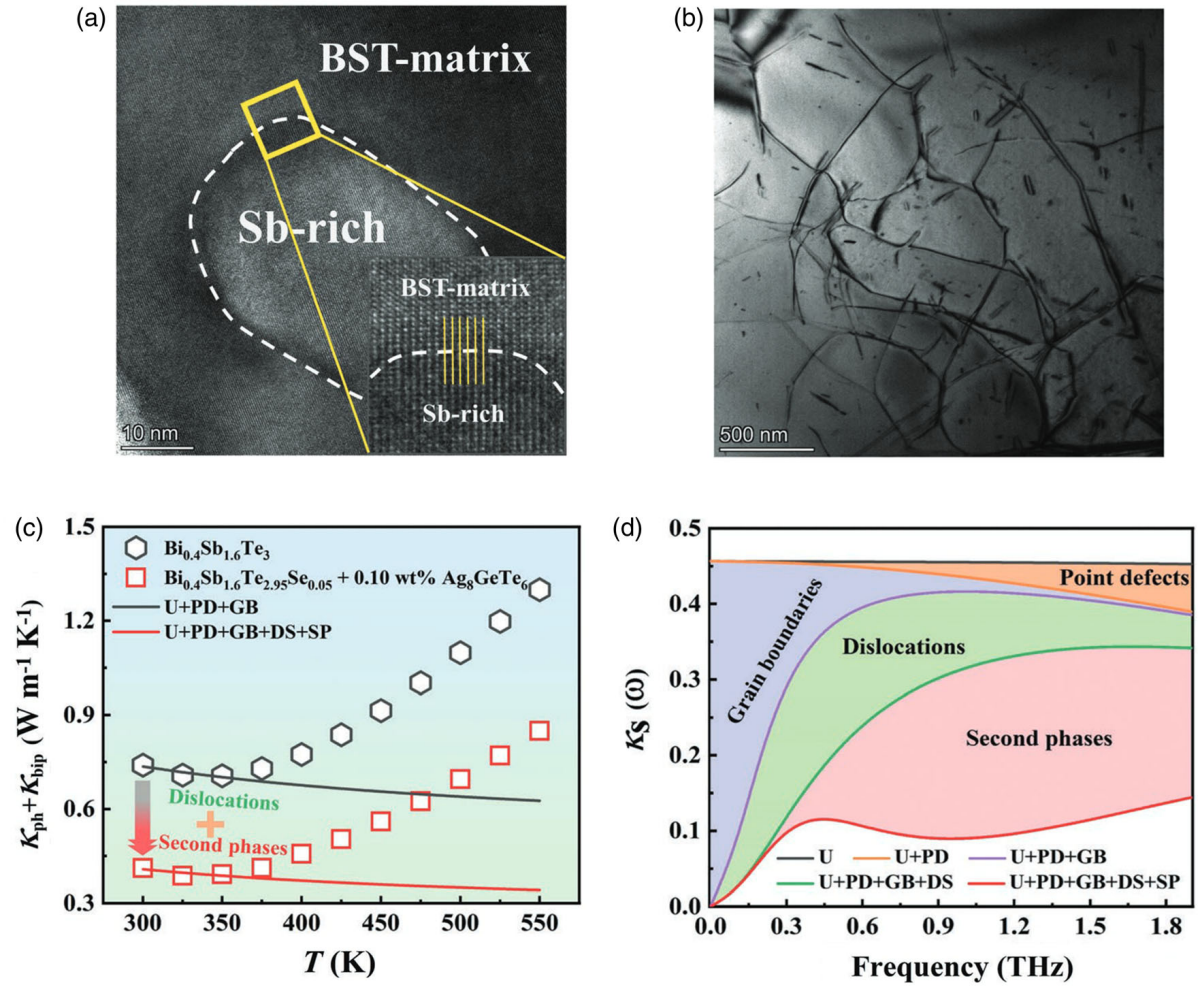
TEM images of a) an Sb‐rich precipitate and its interface with matrix and b) dislocation arrays in Bi_0.4_Sb_1.6_Te_2.95_Se_0.05_ incorporated with 0.10 wt% Ag_8_GeTe_6_. c) Experimental and calculated data (without regard to bipolar diffusion) of the samples after considering dislocations and second phases. d) Frequency‐dependent spectral lattice thermal conductivity of Bi_0.4_Sb_1.6_Te_2.95_Se_0.05_ incorporated with 0.10 wt% Ag_8_GeTe_6_ calculated by the Debye–Callaway model. Reproduced with permission.^[^ ^91^ ^]^ Copyright 2023, Wiley‐VCH GmbH.

The incorporation of low thermal conductivity nanoinclusions is a strategy that has emerged in recent years, literatures of which are summarized in **Table** **3**. It is worth noting that not only did the *ZT* value generally improve overall, but PF and *κ*_L_ were also significantly optimized in all cases. Jiang's group has conducted an extensive and in‐depth exploration of this method.^[^ ^86^, ^87^, ^88^, ^89^, ^90^, ^91^, ^92^, ^93^ ^]^ However, their work focused on p‐type (Bi,Sb)_2_Te_3_, possibly because doping of Ag or Cu is more beneficial to p‐type (Bi,Sb)_2_Te_3_. They screened a number of compounds with low thermal conductivity that could improve the TE performance of p‐type (Bi,Sb)_2_Te_3_, such as AgSbTe_2_,^[^ ^86^ ^]^ AgBiSe_2_,^[^ ^85^ ^]^ Cu_8_GeSe_6_,^[^ ^89^ ^]^ and so on. These compounds exhibit extremely low *κ*_L_ owing to strong anharmonicity in chemical bonds. By utilizing single‐substance powders of Bi, Sb, Te and powders of compounds with low thermal conductivity as raw materials, they prepared alloys using rocking‐melting and hot‐pressing techniques. Consequently, all these studies reported a homogeneous distribution of the added element. However, most works did not observe nanoinclusions containing the introduced elements; instead, Sb‐rich nanoinclusions were commonly found across these studies which may be attributed to substituted doping of Ag/Cu leading to Sb precipitation (**Figure** **7**a).^[^ ^91^ ^]^ Simultaneously, the doping of Ag/Cu may induce a significant change in carrier concentration, which can be adjusted by introducing another element for doping purposes. Therefore, due to the suppression of the bipolar effect, the peak *ZT* values generally shift toward higher temperatures (Table 3).^[^ ^91^ ^]^ The introduction of multiple elements for doping induces various point defects that further contribute to the formation of dislocations (Figure 7b).^[^ ^91^ ^]^ According to the Debey–Callaway model, both introduced substituted defects and dislocation arrays along with Sb‐rich nanoprecipitates contribute significantly to phonon scattering mechanisms within the material system studied here (Figure 7c,d).^[^ ^91^ ^]^ Dense dislocation arrays combined with nanoinclusions create mismatched phonon modes that strongly scatter phonons having short and medium mean free paths thereby impeding heat flow throughout the material system effectively. Moreover, the abundance of Sb‐rich nanoprecipitates may prove superior compared to dispersive point defects for reducing *κ*_L_ near room temperature. Additionally, the coherent interface between Sb‐rich precipitate and matrix material facilitates easy passage for charge carriers, making the interface introduced by Sb‐rich precipitate a successful example of interface engineering.

**Table 3:** Performance enhancement of bismuth telluride incorporated with some different metallic compounds with low thermal conductivity. (The data have been extracted from the figures, and may differ from the textual descriptions provided by the authors).

Type	Matrix	Nanoinclusion	Size [nm]	Increase in PF [%]	Decrease in *κ*_L_ [%]	Peak *ZT* before nanocomposite	Peak *ZT* after nanocomposite	Increase in peak *ZT* [%]	Year and references
P	Bi_0.48_Sb_1.52_Te_3_	AgBiSe_2_	–	15.8	13.8	0.89 at 300 K	1.06 at 375 K	18.8	2021^[^ ^85^ ^]^
P	Bi_0.48_Sb_1.52_Te_3_	AgSbTe_2_	–	26.3	30.3	0.81 at 325 K	1.14 at 325 K	40.1	2021^[^ ^86^ ^]^
P	Bi_0.48_Sb_1.52_Te_3_	AgCuTe	–	36.7	18.2	0.91 at 325 K	1.25 at 350 K	38.2	2021^[^ ^87^ ^]^
P	Bi_0.48_Sb_1.52_Te_3_	AgSbSe_2_	–	16.2	34.9	0.82 at 300 K	1.16 at 350 K	41.1	2021^[^ ^88^ ^]^
P	Bi_0.5_Sb_1.5_Te_3_	Cu_8_GeSe_6_	–	21.8	15.9	1.02 at 300 K	1.30 at 350 K	27.6	2022^[^ ^89^ ^]^
P	Bi_0.5_Sb_1.5_Te_3_	Cu_2_GeSe_3_	–	25.3	2.4	1.11 at 325 K	1.30 at 350 K	16.9	2023^[^ ^90^ ^]^
P	Bi_0.4_Sb_1.6_Te_3_	Ag_8_GeTe_6_	–	8.8	19.7	1.22 at 350 K	1.29 at 350 K	6.0	2023^[^ ^91^ ^]^
P	Bi_0.4_Sb_1.6_Te_3_	Ag_5_Te_3_	–	11.9	28.5	1.22 at 350 K	1.33 at 400 K	9.2	2023^[^ ^92^ ^]^
P	Bi_0.4_Sb_1.6_Te_3_	CsPbI_3_	<50	31.0	20.6	1.00 at 320 K	1.50 at 398 K	50.0	2024^[^ ^94^ ^]^
P	Bi_0.5_Sb_1.5_Te_3_	CdS	1	46.0	14.1	0.97 at 325 K	1.47 at 350 K	51.5	2024^[^ ^93^ ^]^
N	Bi_2_Te_2.6_Se_0.4_	CdS	1	11.0	15.3	0.95 at 425 K	1.08 at 400 K	13.9	2024^[^ ^93^ ^]^

Among the research of Jiang's group, the maximum decreasing amplitude was observed in Bi_0.48_Sb_1.52_Te_3_–*x* wt% AgSbSe_2_ composites.^[^ ^88^ ^]^ In this study, the *κ*_L_ at 300 K decreased by 57% from 0.95 to 0.41 W m^−1^ K^−1^ for the pristine Bi_0.48_Sb_1.52_Te_3_, and further decreased to 0.37 W m^−1^ K^−1^ at a temperature of 350 K. However, the incorporation of AgSbSe_2_ resulted in low Seebeck coefficient and high electronic thermal conductivity due to the high carrier concentration, which limited the increase in *ZT* value at this incorporation amount. Considering that doping with Ag/Cu and Se can lead to opposite variations in carrier concentration, it is possible to achieve higher TE performance by incorporating compounds with low thermal‐conductivity containing Ag/Cu and subsequently doping Se to further regulate the carrier concentration. In fact, Jiang et al. did adopt this approach in their recent work, and obtained lower *κ*_L_ and higher *ZT* value. For the Bi_0.4_Sb_1.6_Te_2.95_Se_0.05_–0.10 wt% Ag_8_GeTe_6_ sample,^[^ ^91^ ^]^ the *κ*_L_ eventually decreased to 0.34 W m^−1^ K^−1^ at 550 K while simultaneously achieving an upgraded the peak *ZT* value of 1.53 at 350 K.

**Figure 8:**
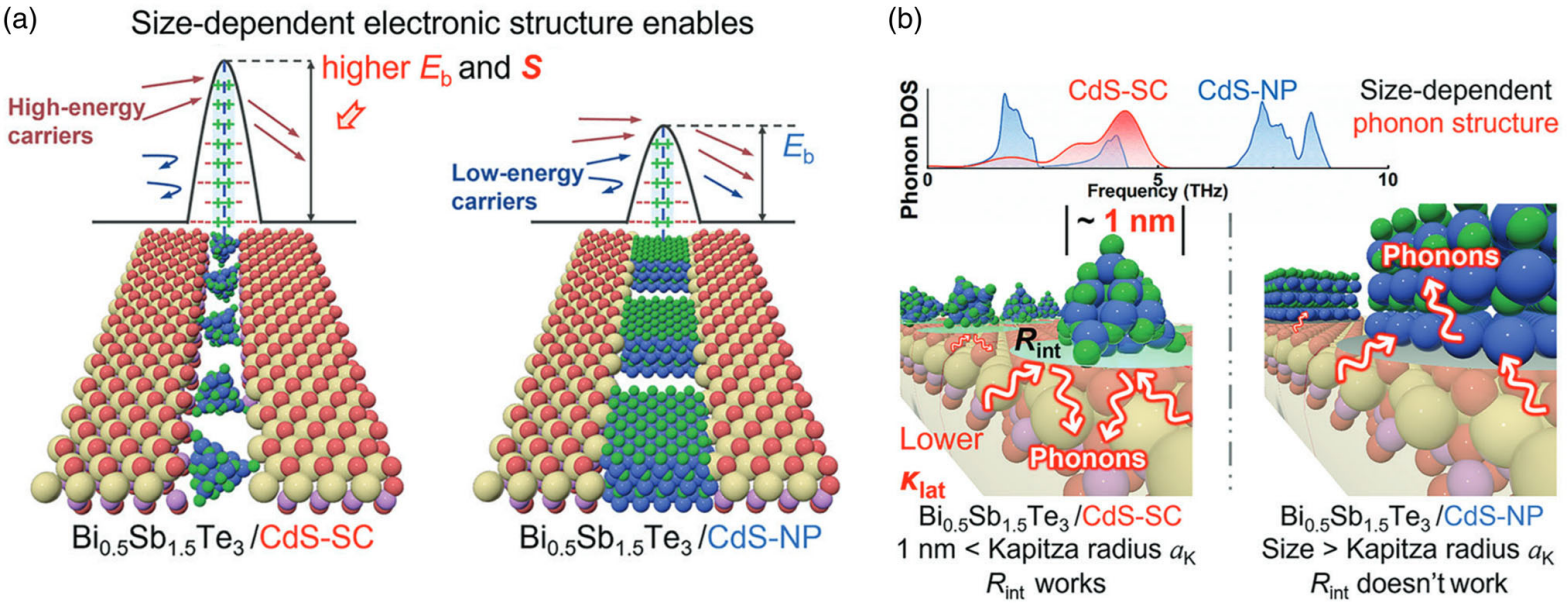
Schematic diagram of the advantages of the CdS supertetrahedron cluster (CdS‐SC, ≈1 nm) in modulating a) Seebeck coefficient and b) *κ*_L_ for the Bi_0.5_Sb_1.5_Te_3_ (BST) material compared with the conventional CdS nanoparticle (CdS‐NP, tens of nm). Reproduced with permission.^[^ ^93^ ^]^ Copyright 2024, Wiley‐VCH GmbH.

Although compounds with low thermal conductivity have been proven effective in enhancing the TE performance of bismuth telluride, their working mechanism is commonly attributed to the doping effect. However, it should be noted that these compounds themselves also contribute significantly to improving TE performance, and this enhancement becomes more pronounced as their size decreases. Xu et al. selected a 1 nm‐sized CdS supertetrahedron cluster as the nanoinclusion.^[^ ^93^ ^]^ To ensure uniform dispersion of CdS without reacting with the bismuth telluride matrix, acetonitrile and acetone were used as solvents instead of ball milling. Consequently, most of the CdS supertetrahedron clusters were distributed near the grain boundaries in bismuth telluride, leading to engineered interfaces, although a small amount of Cd and S was inevitably doped into the matrix. The incorporated CdS supertetrahedron clusters exhibited a strong energy‐filtering effect on charge carrier transport due to their size‐related electronic structure and scattering mechanism (**Figure** **8**a). Additionally, high thermal resistance was contributed by these 1 nm‐sized CdS supertetrahedron clusters because they are smaller than Kapitza radius (Figure 8b). Due to the significant contribution made by these small‐sized nanoinclusions on charge carrier and phonon transport, peak *ZT* values for p‐type Bi_0.5_Sb_1.5_Te_3_ and n‐type Bi_2_Te_2.6_Se_0.4_ improved to 1.47 at 350 K and 1.08 at 400 K respectively. Similar phenomena can be observed when incorporating Zn_4_In_16_S_35_.^[^ ^93^ ^]^ Furthermore, if these compounds with low thermal conductivity possess suitable electronic structures themselves, simply avoiding reaction with the matrix will lead to significant improvement in TE performance; this has been successfully achieved through the incorporation of nanoscale perovskite CsPbX_3_ (X = Cl, Br, or I) (≈50 nm).^[^ ^94^ ^]^ Attributed to the special electronic structure and low thermal conductivity of CsPbI_3_, along with the resulting dislocations, lattice distortions, and point defects, a peak *ZT* of 1.5 at 398 K was obtained with a high average *ZT* of 1.4 from room temperature to 523 K. For the works mentioned above, three repeated measurements were conducted by the authors to verify the stability; however, it is recommended to assess long‐term stability during subsequent investigations due to the potential reactivity between these compounds and the matrix material.

Therefore, it can be concluded that if the elements constituting a compound are ascertained to enhance TE performance upon doping into the matrix of bismuth telluride, and concurrently, the compound exhibits a low *κ*_L_, employing this compound for incorporation harbors the considerable potential for substantial augmentation of TE performance. Moreover, this approach shows promise in further enhancing TE performance through doping.

### 4.4 Low‐Dimensional (1D and 2D) Materials

The incorporated nanoinclusions mentioned above can be considered as 0D nanoparticles. However, incorporating nanoinclusions of varying dimensionalities into bismuth telluride leads to a spectrum of effects on TE performance.^[^ ^95^ ^]^ Compared with the 0D nanoparticles, the incorporation of 1D and 2D materials exhibits significant advantages in scattering phonons owing to the extended interfaces. Moreover, these materials may beneficially modulate charge carrier dynamics, thereby augmenting the electrical transport properties. The incorporation of 1D and 2D materials has exhibited considerable promise in the advancement of TE performance. Although numerous works have focused on incorporating 1D and 2D materials, they have been limited to a narrow range of substances possibly due to the early stage of the research in this field. Some cases that demonstrate improved TE performance by incorporating 1D and 2D materials into bismuth telluride are summarized and presented in **Table** **4**. While electrical transport properties were not necessarily optimized in these cases, *κ*_L_ was generally reduced. This is likely because the regulation of electrical transport is more susceptible to dispersion, and the addition of interfaces through incorporating 1D and 2D materials can effectively enhance phonon scattering.

**Table 4:** Performance enhancement of bismuth telluride incorporated with some different low‐dimensional materials. (The data have been extracted from the figures, and may differ from the textual descriptions provided by the authors).

Type	Matrix	Nanoinclusion	Size	Increase in PF [%]	Decrease in *κ*_L_ [%]	Peak *ZT* before nanocomposite	Peak *ZT* after nanocomposite	Increase in peak *ZT* [%]	Year and references
P	Bi_0.5_Sb_1.5_Te_3_	Expanded graphene	0.5–10 μm	27.5	41.3	0.78 at 329 K	1.13 at 359 K	44.1	2015^[^ ^103^ ^]^
P	Bi_0.4_Sb_1.6_Te_3_	Graphene nanosheets	100–200 nm	−14.9	28.8	1.42 at 395 K	1.54 at 440 K	8.5	2016^[^ ^101^ ^]^
P	Bi_0.5_Sb_1.5_Te_3_	Graphene oxide	–	−3.0	11.5	0.80 at 348 K	0.91 at 398 K	13.7	2016^[^ ^113^ ^]^
P	Bi_0.48_Sb_1.52_Te_3_	Graphene	–	6.3	41.0	0.90 at 320 K	1.25 at 320 K	39.1	2016^[^ ^100^ ^]^
P	Bi_0.36_Sb_1.64_Te_3_–1 wt% Te	Reduced graphene oxide	–	14.3	12.7	1.01 at 393 K	1.16 at 393 K	14.6	2017^[^ ^114^ ^]^
P	Bi_0.4_Sb_1.6_Te_3_	Graphene	–	17.6	40.4	1.09 at 423 K	1.26 at 423 K	15.6	2017^[^ ^99^ ^]^
P	Bi_0.5_Sb_1.5_Te_3_	Graphene nanoplates	1–4 nm	−15.7	3.2	1.04 at 419 K	1.21 at 371 K	16.3	2019^[^ ^115^ ^]^
P	Bi_0.4_Sb_1.6_Te_3_	Ti_3_C_2_T_*x*_ nanosheets	Thickness of 4 nm	6.3	26.1	1.15 at 373 K	1.30 at 398 K	13.2	2020^[^ ^104^ ^]^
P	Bi_0.5_Sb_1.5_Te_3_	Graphene	–	−6.1	9.5	0.89 at 350 K	0.93 at 350 K	4.8	2021^[^ ^111^ ^]^
N	Bi_2_Te_3_	Graphene quantum dots	<20 nm	−29.5	26.9	0.36 at 449 K	0.46 at 448 K	25.2	2017^[^ ^116^ ^]^
N	Bi_2_Te_3_	Graphene nanoplates	1–4 nm	128.5	55.7	0.11 at 326 K	0.55 at 493 K	412.4	2019^[^ ^117^ ^]^
N	Bi_2_Te_2.7_Se_0.3_	Graphene nanoplatelets	–	25.1	30.8	0.68 at 403 K	0.92 at 403 K	35.3	2021^[^ ^118^ ^]^
N	Bi_2_Te_2.5_Se_0.5_	Graphene oxide		6.0	35.1	0.86 at 473 K	1.03 at 473 K	19.7	2022^[^ ^102^ ^]^
N	Bi_2_Te_3_	MWCNTs	Length: 2–3 μm, diameter: 20 nm	16.7	29.6	0.52 at 448 K	0.78 at 423 K	46.6	2015^[^ ^109^ ^]^
N	Bi_2_Te_3_	SWCNTs	Length: 1–10 μm, diameter: 2–10 nm	131.2	55.1	0.09 at 304 K	0.52 at 419 K	464.8	2017^[^ ^106^ ^]^
N	Bi_2_Te_3_	MWCNTs	–	12.3	41.7	0.24 at 328 K	0.36 at 333 K	50.2	2017^[^ ^105^ ^]^

Among the research on incorporating bismuth telluride with 1D and 2D materials, a significant focus has been placed on incorporating it with 2D materials, primarily influenced by the high research interest in graphene. In fact, most research in this area has concentrated on graphene and its derivatives, including single‐layer graphene, multilayer graphene, reduced graphene oxide, and so on. These investigations trace back to 2010 when the Nobel Prize in Physics was awarded for the discovery of graphene.^[^ ^96^ ^]^ Hence, it is difficult not to associate the motivation for incorporating graphene with the popularity surrounding this prestigious award. Furthermore, owing to its relatively high thermal conductivity (the single layer of graphene can even reach 5000 W m^−1^ K^−1^),^[^ ^97^ ^]^ uniform distribution of graphene throughout the matrix is crucial for enhancing TE performance. Otherwise, the agglomerated graphene may adversely affect the *κ*_L_ of incorporated bismuth telluride.

Due to the limited variety of 2D materials available for incorporation, there have been numerous research efforts focused on a small number of graphene derivatives, yielding widely varying results. The TE performance of the same incorporated material may either improve or degrade, largely due to differences in the dispersion processes employed. Generally, two primary methods are employed for dispersing 2D materials: mechanical mixing of 2D materials with raw material powder and utilization of chemical or solvothermal methods to synthesize bismuth telluride and disperse 2D materials in solution. However, the mechanical mixing process may result in a reduction in nanosheet size, particularly when using high‐speed ball milling, which consequently makes it challenging to prevent agglomeration. Conversely, chemical or solvothermal methods can maintain desired nanosheet sizes but present a significant drawback due to the potential introduction of unexpected residual impurities that could considerably compromise TE performance.

**Figure 9:**
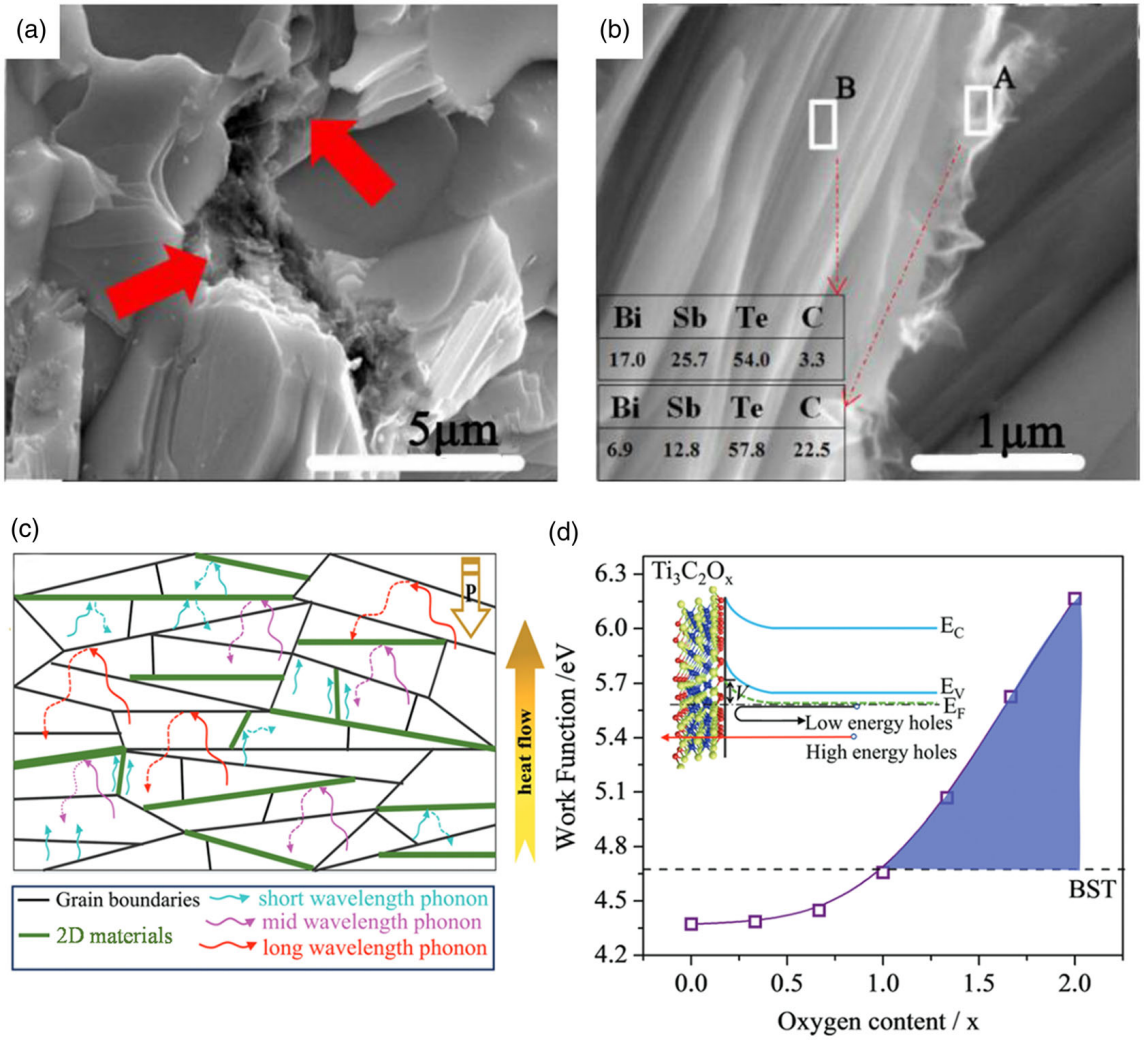
a,b) SEM images for the Bi_0.48_Sb_1.52_Te_3_ sample incorporated with 0.1 wt% graphene.^[^ ^100^ ^]^ c) Schematic diagram of the phonon transport processes in the bismuth telluride incorporated with 2D materials.^[^ ^104^ ^]^ d) The work function of Ti_3_C_2_T_*x*_ as a function of O content. The inset shows the equilibrium band alignment at the interface of Bi_0.5_Sb_1.5_Te_3_ and oxygen‐terminated Ti_3_C_2_T_*x*_ with work function falling in the violet area. Reproduced with permission.^[^ ^104^ ^]^ Copyright 2019, WILEY‐VCH Verlag GmbH & Co. KGaA, Weinheim.

In general, the incorporation of various graphene derivatives has shown potential for enhancing the TE performance of bismuth telluride. As early as 2010, Li et al. incorporated graphene into Bi_2_Te_3_ through a solid‐state reaction.^[^ ^96^ ^]^ However, they solely mixed and compacted the raw materials before sintering without utilizing a graphene dispersion strategy. Although the prepared bulks exhibited smaller grain sizes compared to those without graphene addition, no characterization of graphene dispersion was performed. The introduction of graphene led to a reduction in thermal conductivity, resulting in an increase in *ZT* value from 0.3 to 0.45. On the other hand, Liang et al. prepared a composite material of bismuth telluride with dispersed graphene using a chemical method.^[^ ^98^ ^]^ They added graphene to the solvent during the reaction process, generating a mixed powder which was subsequently consolidated into bulk samples using spark plasma sintering (SPS). It was observed that the incorporation of excessive amounts of graphene may cause agglomeration issues, leading to an increase in electrical resistivity and deterioration of *ZT* values. A similar phenomenon was also noted in the study conducted by Zhang et al. where they utilized ball milling to mix element powder and graphene, followed by high‐pressure‐and‐high‐temperature synthesis and high‐pressure sintering.^[^ ^99^ ^]^ By incorporating 0.1 wt% graphene, the peak *ZT* value of the sample increased from 1.09 to 1.26 at 423 K. In fact, if performed correctly, hand grinding can also yield uniform dispersion of graphene. Xie et al. employed a rocking melting method to prepare Bi_0.48_Sb_1.52_Te_3_ ingots with graphene, which were then manually ground into fine powders before being further sintered using SPS.^[^ ^100^ ^]^ The presence of graphene as floccules at grain boundaries (**Figure** **9**a,b) enhanced the *ZT* value from 0.90 to 1.25 primarily by reducing *κ*_L_. However, similar to other works, optimal performance was achieved when incorporating only 0.05 wt% of graphene; higher amounts resulted in a significant decrease in electrical conductivity and conversely increased *κ*_L_, which was probably due to graphene agglomeration. Hence, the incorporation amount of graphene is a pivotal factor influencing agglomeration, necessitating precise control for ensuring a positive effect.

Although graphene, graphene oxide, and reduced graphene oxide exhibit distinct properties, they all demonstrate similar effects when incorporated into bismuth telluride. However, the highest reported performance for p‐type bismuth telluride was achieved by integrating graphene nanosheets. The peak *ZT* value of Bi_0.4_Sb_1.6_Te_3_ increased from 1.34 at 400 K to 1.54 at 440 K with the addition of only 0.4 vol%, primarily due to a significant reduction in *κ*_L_.^[^ ^101^ ^]^ For n‐type bismuth telluride, optimal performance was attained through the incorporation of graphene oxide;^[^ ^102^ ^]^ however, excess Te was added during the preparation process to facilitate liquid phase sintering which may also contribute to high TE properties in this case. By incorporating 1 wt% graphene oxide into Bi_2_Te_2.5_Se_0.5_, the peak *ZT* value increased to 1.03 at 473 K. However, both of the aforementioned investigations lacked sufficient attention to the state of graphene, and the added graphene may consist of a mixture of single and multilayer forms. Adjusting the state of graphene can potentially further enhance its TE performance. Suh et al. investigated the impact of extended graphene on the TE performance of p‐type Bi_0.5_Sb_1.5_Te_3_, revealing that using extended graphene can significantly reduce *κ*_L_ while increasing peak *ZT* by 45%.^[^ ^103^ ^]^ Therefore, optimizing treatment methods for graphene may lead to even greater improvements in TE performance.

In recent years, there has been an increasing focus on studying bismuth telluride incorporated with graphene oxide or reduced graphene oxide compared to ordinary graphene due to its relatively higher cost compared to graphene oxide or reduced graphene oxide materials. However, there is still a lack of comprehensive understanding regarding the mechanism differences among these materials in regulating the electrical and thermal transport properties of bismuth telluride; thus, further investigation is warranted. Nevertheless, it remains consistent that these 2D materials exhibit high thermal resistance when uniformly distributed at the grain boundaries. These newly formed interfaces contribute to significant scattering of mid‐frequency phonons, consequently reducing the *κ*_L_ of the composite (Figure 9c).

In addition to graphene and its derivatives, active exploration is also underway for the incorporation of other 2D materials. One notable example is Ti_3_C_2_T_*x*_ in MXene. Lu et al. fabricated Bi_0.5_Sb_1.5_Te_3_ incorporated with MXene (Ti_3_C_2_T_*x*_) using a self‐assembly strategy.^[^ ^104^ ^]^ They discovered that the work function of Ti_3_C_2_T_*x*_ can be influenced by the oxygen content determined by the surface configuration (=O, —OH, or —F), resulting in an energy‐filtering effect when incorporated into Bi_0.5_Sb_1.5_Te_3_ (Figure 9d). Combined with significant phonon scattering that suppresses thermal conductivity, this leads to an improvement in *ZT* value from 1.15 to 1.3 and achieves an average *ZT* value of 1.23 from 300 to 475 K, ensuring a high energy conversion efficiency of 7.8% for the prepared device. Therefore, there are still numerous unexplored 2D materials for the incorporation of bismuth telluride. These materials, including graphene, can also adjust the work function and thermal conductivity by modifying their functional groups, which holds great potential for significantly improving the TE performance of bismuth telluride.

**Figure 10:**
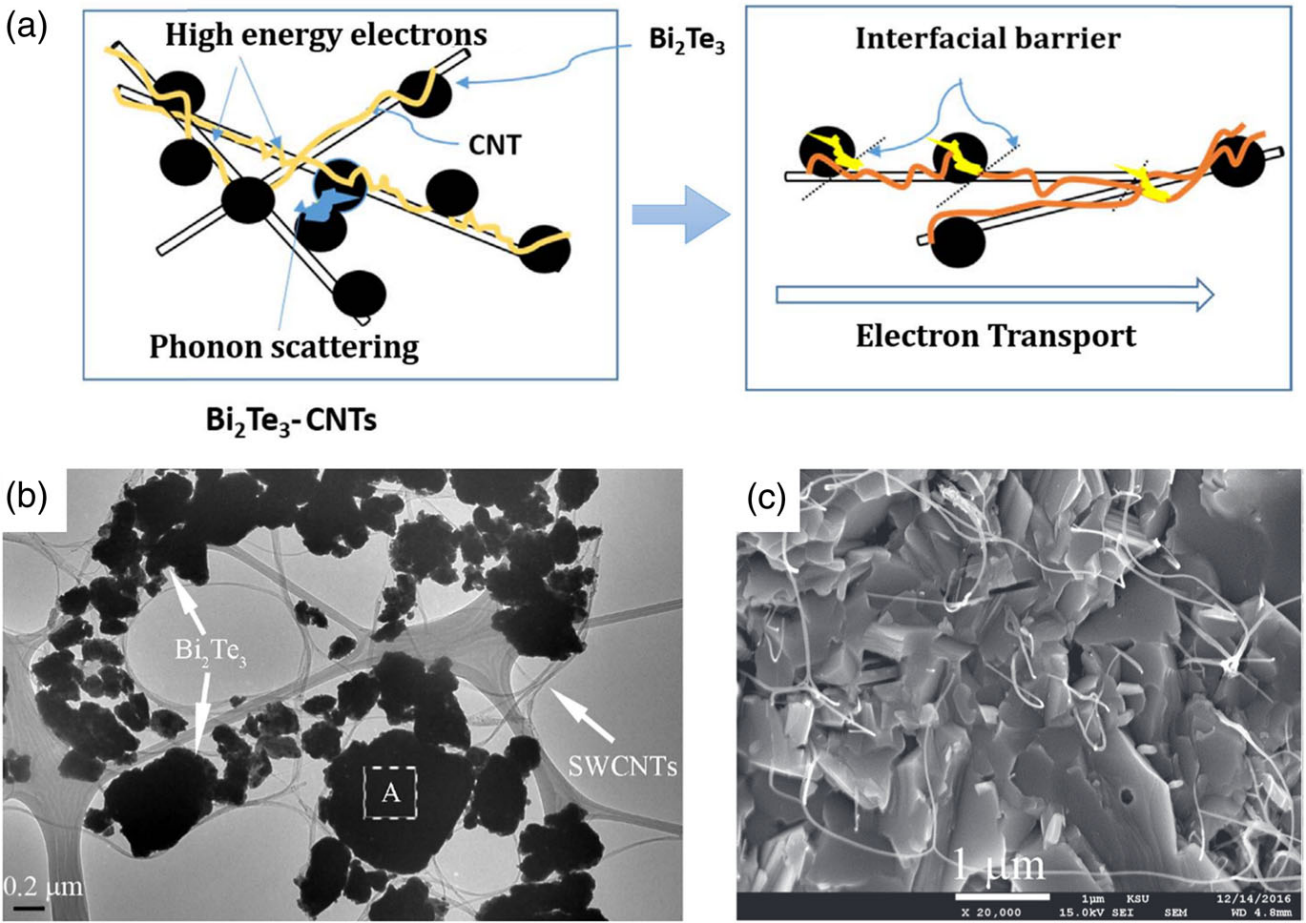
a) Schematic diagram of charge carrier and phonon transport mechanism in the Bi_2_Te_3_ incorporated with CNTs Reproduced with permission.^[^ ^105^ ^]^ Copyright 2017, Elsevier Ltd. and Techna Group S.r.l. b) TEM image of the mixture of Bi_2_Te_3_ powder and 1.0 vol% SWCNTs.^[^ ^106^ ^]^ c) SEM image of the fracture surface of Bi_2_Te_3_ incorporated with 0.5 vol% SWCNT. Reproduced with permission.^[^ ^106^ ^]^ Copyright 2017, IOP Publishing Ltd.

Correspondingly, carbon nanotubes (CNTs) are the most crucial 1D materials employed for incorporation, including single‐walled CNTs (SWCNTs) and multiwalled CNTs (MWCNTs). It is hypothesized that the uniform dispersion of CNTs at grain boundaries can generate numerous heterogeneous interfaces and enhance phonon scattering. When exhibiting metallic properties, both SWCNTs and MWCNTs can establish carrier transport channels due to their high electrical conductivity. The schematic representation of the mechanism for the influence of CNTs on charge carrier and phonon transport in bismuth telluride is illustrated in **Figure** **10**a.^[^ ^105^ ^]^ The uniform mixture of CNTs and bismuth telluride powder can be achieved through simple ball milling (Figure 10b), while the structure of the hypothesized model can be obtained after sintering (Figure 10c).^[^ ^106^ ^]^ Nevertheless, as SWCNTs may exhibit semiconductor characteristics with low electrical conductivity attributed to chirality rather than metal properties, most studies have utilized MWCNTs for incorporating bismuth telluride.

However, the observed effect does not align with expectations. The degree of improvement shown by different studies varies greatly, possibly due to variations in CNT dispersion. For both p and n types, the highest performance was achieved through the incorporation of MWCNTs using the ball milling method as reported by Oh's group. By employing this method, the peak *ZT* value of p‐type (Bi_0.2_Sb_0.8_)_2_Te_3_ increased from 1.03 to 1.47 while n‐type Bi_2_(Te_0.9_Se_0.1_)_3_ increased from 0.88 to 0.98, respectively;^[^ ^107^, ^108^ ^]^ however, the increase in n‐type bismuth telluride was relatively small compared to p‐type material enhancement. The decrease in electrical resistivity was not significant enough to indicate a possible absence of formed carrier transport channels. Besides, p‐type (Bi_0.2_Sb_0.8_)_2_Te_3_ even demonstrated an increased electrical resistivity compared to n‐type Bi_2_(Te_0.9_Se_0.1_)_3_. The improvement in thermal conductivity reduction contributed significantly, suggesting that phonon scattering induced by introduced MWCNTs interfaces might be more intrinsically responsible for improvements. It should be noted that these interfaces can also enhance charge carrier scattering. As observed by scanning electron microscopy (SEM), these introduced MWCNTs were found to be shortened during collisions with steel balls, potentially hindering the effective formation of carrier transport channels.

In other studies, particularly in the chemical synthesis of bismuth telluride and dispersion of CNTs in solution, a larger proportion of enhancements have been achieved in the *ZT* value. However, due to the limited intrinsic performance of bismuth telluride, the resulting *ZT* values remain relatively low. For instance, Kim et al. employed a solution‐based approach to incorporate CNTs into Bi_2_(Se,Te)_3_ and observed a 78% increase in peak *ZT* value from 0.53 to 0.93 compared to Bi_2_Te_3_ without CNTs.^[^ ^109^ ^]^ Similarly, Kumar et al. using a similar method, incorporated MWCNTs into Bi_2_Te_3_ and achieved a 45% enhancement in peak *ZT* value from 0.24 to 0.36.^[^ ^105^ ^]^ Furthermore, they also noted an increase in electrical conductivity and a decrease in *κ*_L_ after incorporating MWCNTs. These findings suggest that improved electrical transport properties may indeed be attributed to the uniform dispersion of MWCNTs. Moreover, it is undeniable that the interface introduced by CNT incorporation does facilitate phonon scattering regardless of the incorporation method employed. However, it is important to explore alternative strategies for achieving more uniform dispersion for CNTs.

## 5 Summary and Outlook

**Figure 11:**
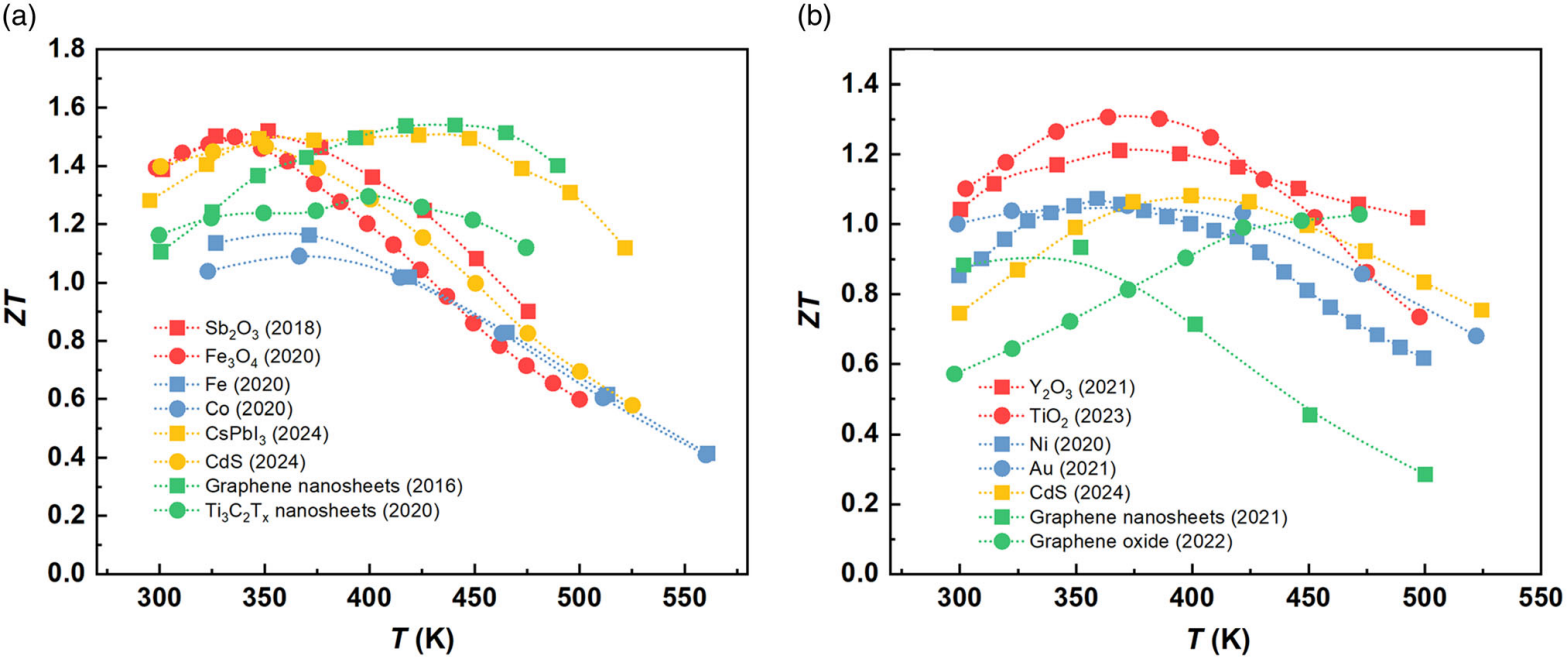
Temperature dependence of *ZT* for a) p‐type and b) n‐type bismuth telluride incorporated with several representative nanoinclusions of nonmetallic hard nanoparticle (red),^[^ ^53^, ^56^, ^71^, ^72^ ^]^ metallic nanoparticle (blue),^[^ ^76^, ^77^, ^81^ ^]^ compounds with low thermal conductivity (yellow),^[^ ^93^, ^94^ ^]^ and low‐dimensional materials (green).^[^ ^101^, ^102^, ^104^, ^111^ ^]^

In summary, the incorporation of nanoinclusions into bismuth telluride represents a prevalent strategy for enhancing TE performance while concurrently improving mechanical properties. Numerous studies have been conducted based on this approach, encompassing various types of nanoinclusions such as nonmetallic hard nanoparticles, metallic nanoparticles, compounds with low thermal conductivity, and low‐dimensional materials. The temperature dependence of *ZT* values of several representative cases ranking high in TE performance for each type of nanoinclusion is shown in **Figure** **11**. For p‐type bismuth telluride, the incorporation of nonmetallic hard nanoparticles, compounds with low thermal conductivity, and low‐dimensional materials can all achieve high *ZT* values. In contrast, for n‐type bismuth telluride, incorporating nonmetallic hard nanoparticles shows the optimum effect. The pivotal role played by these nanoinclusions lies in their ability to introduce additional interfaces that enhance phonon scattering. Furthermore, the band bending induced by metal–semiconductor or semiconductor–semiconductor contacts at interfaces can potentially induce selective carrier scattering (energy‐filtering effect), along with multiple scattering effects caused by interface defects, thereby augmenting the Seebeck coefficient.

Among the materials discussed, nonmetallic hard nanoparticles have a well‐established research history and numerous associated reports. They not only function as effective phonon scattering centers but also effectively suppress grain growth, thereby enhancing grain boundary scattering. Due to their limited reactivity with bismuth telluride, these hard nanoparticles tend to form interfaces rich in defects, which simultaneously increase phonon scattering and induce multiple scattering effects on carriers. Moreover, the contact between semiconductors may result in an energy‐filtering effect. Both the multiple scattering and energy‐filtering effects possess significant potential for augmenting the Seebeck coefficient. Therefore, a continued comprehensive exploration of nonmetallic hard nanoparticles represents an effective strategy for further enhancing the TE performance of bismuth telluride.

The incorporation of metallic nanoparticles can induce metal–semiconductor contact, thereby enabling energy‐filtering for electrons. Consequently, specific metallic nanoparticles such as Au and Ni have the potential to enhance the Seebeck coefficient of n‐type bismuth telluride. Additionally, ferromagnetic metal nanoparticles below a certain size may exhibit superparamagnetism, leading to multiple scattering effects due to the magnetic drag effect that could further augment the Seebeck coefficient. Moreover, the interaction between metallic nanoparticles and bismuth telluride can influence both the interface contact type and size‐related superparamagnetic effects. Hence, precise control over the reaction between metallic nanoparticles and bismuth telluride is crucial for regulating nanocomposites using metallic nanoparticles.

The enhancement of TE performance in bismuth telluride through the incorporation of compounds of low thermal conductivity primarily arises from doping effects, despite the initial intention being to reduce overall thermal conductivity via composite effects. Most of these compounds typically consist of Ag‐ or Cu‐based Te or Se compounds, and their contribution to improved TE performance is predominantly attributed to doping effects. These findings suggest that a nanocomposite strategy could be pursued by seeking compounds with low thermal conductivity that contain elements capable of enhancing TE performance through doping effects.

Low‐dimensional materials, including 2D and 1D materials such as graphene and its derivatives, as well as CNTs, play a crucial role in the development of nanocomposites. These nanoinclusions exhibit a distinctive characteristic of forming multiple interfaces with bismuth telluride, thereby significantly enhancing phonon scattering. Moreover, their high conductivity suggests the potential to establish continuous carrier transport channels to enhance electrical conductivity; however, due to existing challenges in uniform dispersion, this objective has not yet been realized. Consequently, future research on low‐dimensional materials should prioritize strategies for achieving uniform dispersion.

We have systematically summarized the research context of each type of nanoinclusions, analyzed the key factors contributing to the enhancement of TE performance, and presented significant case studies. Additionally, we have identified the existing challenges associated with each type of nanoinclusions while proposing future directions for further investigation. Based on the aforementioned points, we present a concise overview of current perspectives and propose future research directions for nanocomposite strategy in bismuth telluride: 1) The dispersion uniformity of the nanoinclusions significantly impacts the TE performance of bismuth telluride‐based nanocomposites. The ball milling method exhibits mediocre dispersion uniformity and poses a risk of structural damage to 1D or 2D materials used for nanocomposite. Although the chemical process in solution can achieve better dispersion uniformity, it also carries the potential for introducing unexpected impurities. Therefore, there is an urgent need to develop novel and more uniform dispersion strategies. 2) Currently, the interface issue in nanocomposite research has not received sufficient attention, necessitating the utilization of more advanced techniques to characterize the interface. This will enable differentiation between the enhancement effects on the Seebeck coefficient by diverse nanoinclusions, whether it is attributed to multiple scattering effects resulting from interface defects or band bending caused by contact, thereby preventing any potential misleading for subsequent research. 3) As a commonly employed theory to explain the high Seebeck coefficient of nanocomposites, the discussion about energy‐filtering effect is basically qualitative in current work. Future investigations are encouraged to undertake more comprehensive analyses of this effect. 4) The influence mechanism of different processes and nanoinclusions relative to interface defects needs to be further studied in order to explore new processes for improving the Seebeck coefficient by constructing interface defects to achieve multiple scattering effects. 5) More attention needs to be paid to the reactions between the nanoinclusion and bismuth telluride during the preparation process, as these reactions can significantly impact the interface properties and size of nanoinclusions, thereby further influencing the overall effectiveness of the nanocomposite. 6) The introduction of the nanoinclusions typically exerts an influence on the grain size or defects, leading to a deviation in carrier concentration from the optimal range. Therefore, incorporating element doping into nanocomposites represents a promising approach for achieving enhanced TE performance.

In summary, the incorporation of nanoinclusions is a reliable approach to enhancing the TE performance of bismuth telluride. Despite significant progress achieved in previous studies, further exploration of nanocomposite strategy for bismuth telluride‐based TE materials remains imperative to facilitate their commercialization and widespread application in refrigeration.

## Conflict of Interest

The authors declare no conflict of interest.
